# DNA double-strand break-derived RNA drives TIRR/53BP1 complex dissociation

**DOI:** 10.1016/j.celrep.2022.111526

**Published:** 2022-10-25

**Authors:** Ruth F. Ketley, Federica Battistini, Adele Alagia, Clémence Mondielli, Florence Iehl, Esra Balikçi, Kilian V.M. Huber, Modesto Orozco, Monika Gullerova

**Affiliations:** 1 Sir William Dunn School of Pathology, South Parks Road, Oxford OX1 3RE, UK; 2 Institute for Research in Biomedicine (IRB Barcelona), The Barcelona Institute of Science, and Technology, Baldiri Reixac 10–12, 08028 Barcelona, Spain; 3 Department of Biochemistry and Molecular Biology. University of Barcelona, 08028 Barcelona, Spain; 4 HTBS – Biophysics Group, Evotec (France) SAS, Campus Curie, 195 Route d’Espagne, 31036 Toulouse Cedex, France; 5 Centre for Medicines Discovery, Nuffield Department of Medicine, Oxford OX3 7FZ, UK; 6 Target Discovery Institute, Nuffield Department of Medicine, Oxford OX3 7FZ, UK

**Keywords:** DNA damage, double-strand breaks, TIRR, 53BP1, RNA, hairpin, NHEJ, FISH-PLA, DARTs, dilncRNA

## Abstract

Tudor-interacting repair regulator (TIRR) is an RNA-binding protein and a negative regulator of the DNA-repair factor p53-binding protein 1 (53BP1). In non-damage conditions, TIRR is bound to 53BP1. After DNA damage, TIRR and 53BP1 dissociate, and 53BP1 binds the chromatin at the double-strand break (DSB) to promote non-homologous end joining (NHEJ)-mediated repair. However, the exact mechanistic details of this dissociation after damage are unknown. Increasing evidence has implicated RNA as a crucial factor in the DNA damage response (DDR). Here, we show that RNA can separate TIRR/53BP1. Specifically, RNA with a hairpin secondary structure, transcribed at the DSB by RNA polymerase II (RNAPII), promotes TIRR/53BP1 complex separation. This hairpin RNA binds to the same residues on TIRR as 53BP1. Our results uncover a role of DNA-damage-derived RNA in modulating a protein-protein interaction and contribute to our understanding of DSB repair.

## Highlights

• RNA derived from double-strand breaks (DSBs) can promote TIRR/53BP1 complex dissociation
• RNAPII-transcribed hairpin RNA binds to the same residues on TIRR as 53BP1
• TIRR/53BP1 separate in proximity to the DSB
• Dicer and Drosha are required for TIRR/53BP1 dissociation

## Graphical abstract

**Figure:**
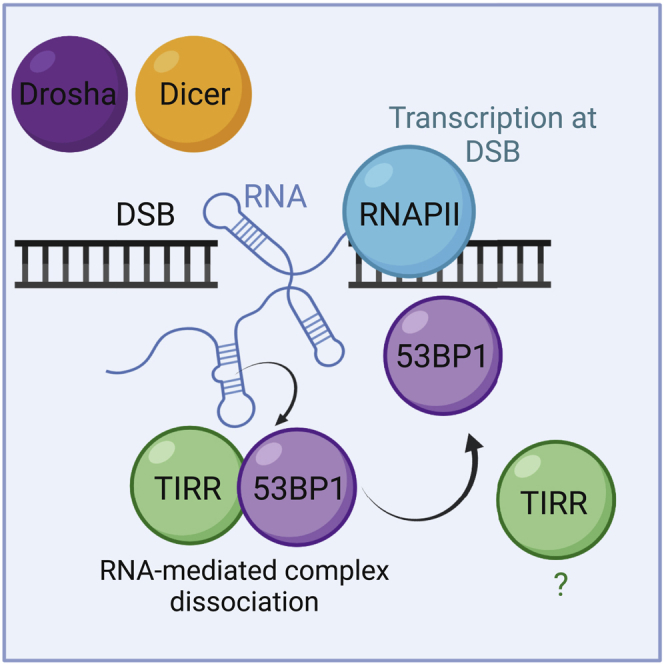

## Teaser

Cells constantly experience different types of DNA damage, including toxic double-strand breaks (DSBs). RNA is an important player in DSB repair. Ketley et al. show that RNA transcribed at DSBs promotes separation of the repair protein 53BP1 from its negative regulator TIRR, contributing to our understanding of DSB repair.

## Introduction

DNA double-strand breaks, where both strands of the DNA double helix are broken, are considered to be one of the most harmful types of DNA damage (Jackson and Bartek, 2009). Double-strand breaks (DSBs) can be repaired by two main pathways, homologous recombination (HR), which requires 5′ → 3′ end resection of the DNA at the break, and non-homologous end joining, which does not require end resection (Panier and Boulton, 2014). p53-binding protein 1 (53BP1) is an anti-end resection repair factor and, in non-damage conditions, is bound by Tudor-interacting repair regulator (TIRR). TIRR, a paralog of the Nudix protein NUDT16, binds to the Tudor domains of 53BP1, masking the H4K20me2 binding site needed for 53BP1 chromatin association after damage. However, when a DSB occurs, TIRR and 53BP1 dissociate, and 53BP1 can then bind to chromatin at the DSB site through recognition of the constitutive H4K20me2 through the Tudor domain and the damage-specific H2AK15Ub through the ubiquitination-dependent recruitment (UDR) motif (Dai et al., 2018; Drané et al., 2017; Wang et al., 2018; Zhang et al., 2017). However, the exact mechanistic details of how TIRR/53BP1 dissociate after DNA damage has occurred remain elusive.

Over the last decade, the importance of various RNA classes, including long non-coding RNAs (lncRNA) and microRNAs (miRNA), in the repair of DSB lesions has been demonstrated (Ketley and Gullerova, 2020). In particular, RNA polymerase II (RNAPII) transcription from the DSB ends has been shown to produce damage-induced lncRNA (dilncRNA) and damage responsive transcripts (DARTs) (Bonath et al., 2018; Burger et al., 2019; Michelini et al., 2017). dilncRNA has been found to have different functions in DSB repair, including in forming RNA:DNA hybrids at the DSB, and can also be processed by the ribonuclease III enzymes Dicer and Drosha into small non-coding RNAs known as DNA-damage RNAs (DDRNAs) (Bonath et al., 2018; Burger et al., 2017, 2019; Francia et al., 2012, 2016; Michelini et al., 2017; Pessina et al., 2019). dilncRNA and DDRNA are also known to play functional roles in DSB repair, including in the recruitment of 53BP1 (Burger et al., 2019; Francia et al., 2016).

TIRR is an RNA-binding protein (Avolio et al., 2018), and interestingly, the mapped RNA-binding site on TIRR overlaps with the site of 53BP1 binding (Botuyan et al., 2018; He et al., 2016), suggesting a potential RNA-dependent mechanism of TIRR/53BP1 dissociation (Botuyan et al., 2018). Furthermore, it has been shown that NUDT16 modified with the 53BP1-binding loop of TIRR can bind 53BP1, and the addition of a nucleotide inosine monophosphate (IMP) can reduce this binding (Botuyan et al., 2018). Here, using a combination of *in vitro* and *in vivo* approaches, we show that TIRR/53BP1 dissociation is promoted by RNAPII transcription and RNA molecules. We further show that TIRR can bind single-stranded RNA and hairpin RNA structures and that hairpin RNA can directly separate the TIRR/53BP1 complex *in vitro* and *in vivo*. Furthermore, we show that TIRR is also recruited to damaged DNA sites; however, recruitment of a mutated TIRR, which cannot bind 53BP1 (K10E), is impaired, suggesting that TIRR and 53BP1 are in proximity to chromatin while still bound as a complex, and their dissociation occurs at the DSB itself. We also develop a technique to study the interactions of proteins with RNA *de novo* transcribed at sequence-specific DSBs; fluorescence *in situ* hybridization proximity ligation assay (FISH-PLA). Using the FISH-PLA technique, we find that TIRR and 53BP1 are in close proximity to DSB-derived RNA, implicating nascent RNA in TIRR/53BP1 dissociation. These results not only uncover a previously uncharacterized mechanism of TIRR/53BP1 dissociation in response to DSBs but also highlight the diverse and crucial roles of RNA in the DNA-damage response. In addition, cancers with HR deficiencies are sensitive to treatment with poly (ADP-ribose) polymerase inhibitors (PARPis) (Noordermeer and van Attikum, 2019). Resistance to PARPis can occur through mechanisms including 53BP1 loss of function, and it has been recently suggested that increased TIRR expression and deregulation of the TIRR/53BP1 interaction could contribute to resistance development (Botuyan et al., 2018; Drané et al., 2017; Wang et al., 2018). Therefore, understanding how the TIRR/53BP1 dissociation is regulated could help understand mechanisms of PARPi resistance and provide new therapeutic opportunities.

## Results

### RNAPII transcription is required for TIRR/53BP1 dissociation

To study the TIRR/53BP1 interaction in non-damage and damage conditions, we utilized a quantitative *in vivo* PLA (Figures S1A and S1B). Using the PLA assay with antibodies against TIRR and 53BP1, we detected the presence of PLA foci in non-damage conditions, indicative of TIRR and 53BP1 interaction. Induction of DSBs using etoposide (Eto) treatment led to a reduced number of PLA foci (Figures S1C and S1D), indicating a reduced interaction of TIRR and 53BP1 in damage conditions, which is in agreement with previous observations that TIRR and 53BP1 dissociate in response to DSBs (Drané et al., 2017). We further confirmed the specificity of the PLA assay using TIRR knockdown by a doxycycline-inducible short hairpin RNA (shRNA) against TIRR (Avolio et al., 2018) and observed that the TIRR/53BP1 PLA foci decrease as expected (Figures S1E and S1F).

**Figure 1:**
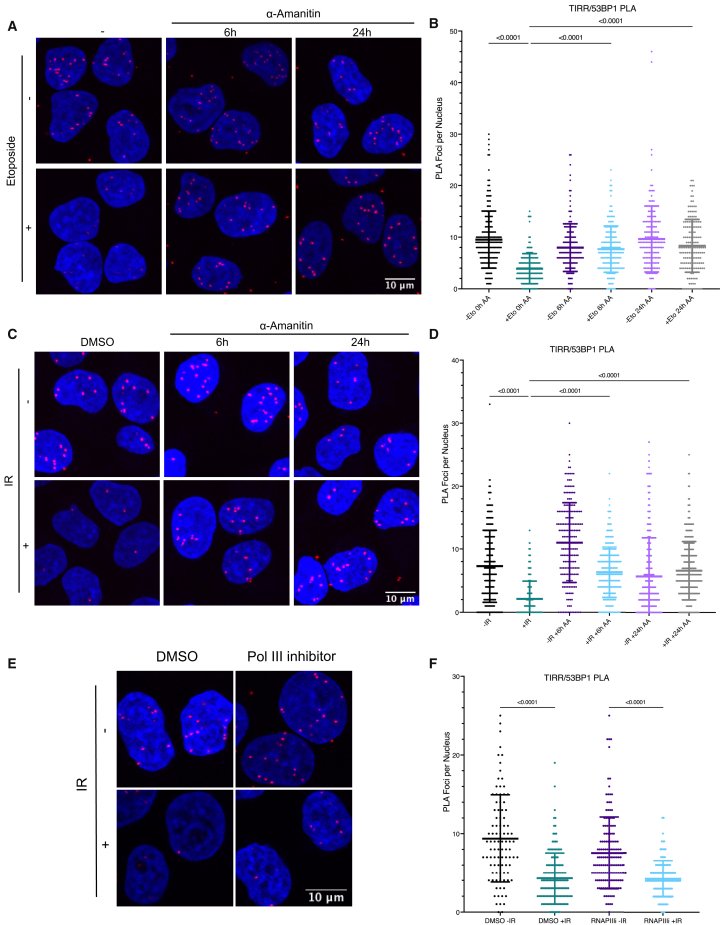
RNA Polymerase II transcription is required for TIRR/53BP1 dissociation (A) PLA of TIRR and 53BP1 with and without etoposide (10 μM, 2 h) in the presence of α-Amanitin (2 μg/mL, 6 or 24 h). n(0 h − Eto) = 187, n(0 h + Eto) = 158, n(6 h − Eto) = 166, n(6 h + Eto) = 173, n(24 h − Eto) = 158, n(24 h + Eto) = 155. (B) Quantification of (A) (mean ± SD, n = 2). (C) PLA of TIRR and 53BP1 with and without IR (10 Gy, 1 h) in the presence of α-Amanitin (2 μg/mL, 6 or 24 h). n(0 h − IR) = 350, n(0 h + IR) = 528, n(6 h − IR) = 196, n(6 h + IR) = 359, n(24 h − IR) = 417, n(24 h + IR) = 317. (D) Quantification of (C) (mean ± sd, n = 2). (E) PLA of TIRR and 53BP1 with and without IR (10 Gy, 1 h) in the presence of an RNA polymerase III (RNAPIII) inhibitor (30 μM for 24 h). n(DMSO − IR) = 94, n(DMSO + IR) = 148, n(RNAPIIIi − IR) = 149, n(RNAPIIIi + IR) = 117. (F) Quantification of (E) (mean ± SD, n = 2).

Since RNAPII RNA transcripts are known to play a key role in DSB repair (Bonath et al., 2018; Burger et al., 2019; Michelini et al., 2017; Wei et al., 2012), we asked whether transcription and RNA transcripts could play a role in TIRR/53BP1 dissociation. We performed the PLA assay in the presence of an RNAPII inhibitor, α-Amanitin (2 μg/mL, 6 or 24 h) (Burger et al., 2019), and observed that TIRR/53BP1 dissociation is impaired when RNAPII transcription is inhibited prior to the induction of DSBs by Eto (10 μM, 2 h) (Figures 1A and 1B) and by ionizing radiation (IR; 10 Gy, 1 h) (Figures 1C and 1D), indicating that RNAPII transcription inhibition prevents TIRR and 53BP1 dissociation in response to DSBs induced by Eto and IR. Additionally, given that RNAPIII transcription has also been shown to transcribe RNA at DSBs (Liu et al., 2021), we also performed PLA in the presence of an RNAPIII inhibitor (ML-60218, 30 μM for 24 h); however, we could not detect any impact of RNAPIII inhibition on TIRR/53BP1 dissociation after IR (Figures 1E and 1F). Collectively, these data suggest that TIRR/53BP1 dissociation after DNA damage is influenced predominantly by RNAPII transcription rather than RNAPIII transcription.

### TIRR binds RNA with hairpin and single-stranded structures

**Figure 2:**
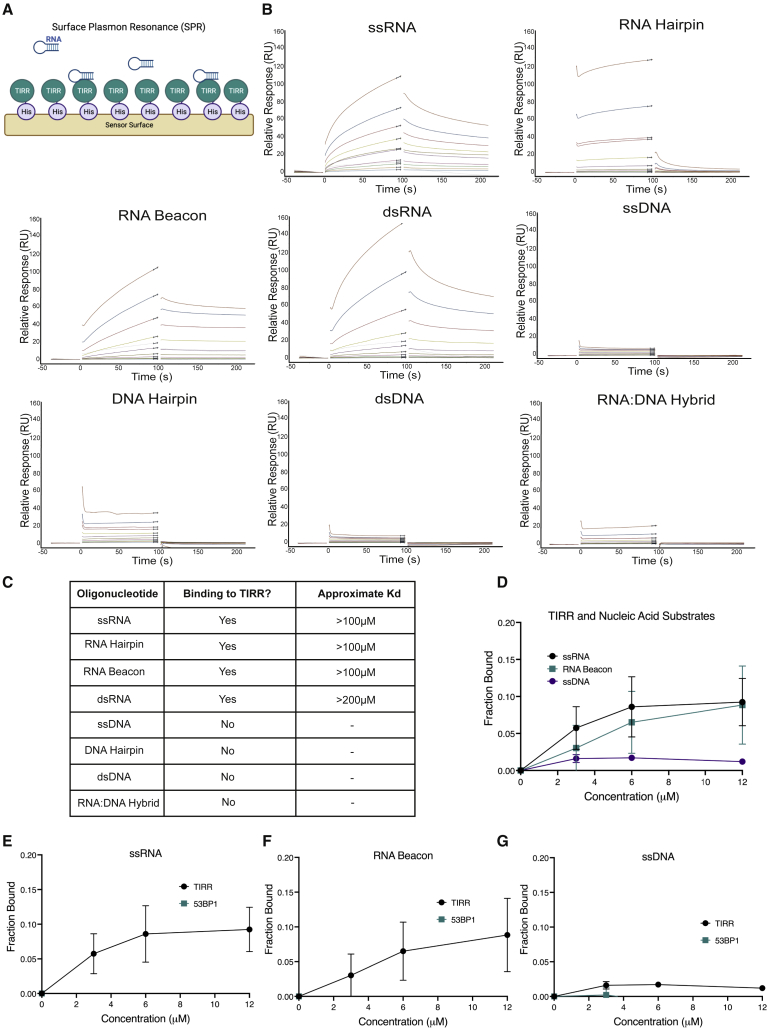
TIRR binds RNA with hairpin and single-stranded structures (A) Principles of the surface plasmon resonance (SPR) assay. His-TIRR is immobilized on a sensor chip surface, increasing concentrations of RNA and DNA substrates are added, and binding is measured. Image was created with BioRender.com. (B) SPR with His-TIRR and (left → right) ssRNA, RNA hairpin, RNA beacon, dsRNA, ssDNA, DNA hairpin, dsDNA, and RNA:DNA hybrid. Increasing concentrations of DNA/RNA added up to 200 μM. (C) Summary of (B). Table showing the calculated binding constant (Kd) from the SPR data. (D) Quantification of EMSAs with ssRNA (n = 4), RNA beacon (n = 3), and ssDNA (n = 3) and increasing concentrations of TIRR. (E) Quantification of EMSAs showing comparison with ssRNA and increasing concentration of TIRR (as shown in D) and 53BP1-Tudor (n = 3) (mean ± SD). (F) Quantification of EMSAs showing comparison with RNA beacon and increasing concentration of TIRR (as shown in D) and 53BP1-Tudor (n = 3) (mean ± SD). (G) Quantification of EMSAs showing comparison with ssDNA and increasing concentration of TIRR (as shown in D) and 53BP1-Tudor (n = 2) (mean ± SD).

TIRR has been shown to be an RNA-binding protein (Avolio et al., 2018), although the RNA-binding specificity of TIRR is not well characterized. RNA can exert its functions in cells in a variety of ways, including by having a specific sequence or a specific secondary structure. With a view to establish the RNA-binding properties of TIRR, we assessed if TIRR binds specific RNA secondary structures. We employed a surface plasmon resonance (SPR) assay (Figure 2A) with immobilized TIRR and different RNA substrates: single-strand RNA (ssRNA), two different RNA hairpins (RNA hairpin and RNA beacon), double-stranded RNA (dsRNA), an RNA:DNA hybrid, and the equivalent DNA structures (Figure S2). We observed that TIRR can bind RNA with ssRNA and RNA hairpins structures, with an approximate weak binding affinity (Kd) of around 100 μM and with a lower affinity for dsRNA (∼200 μM). We could not detect comparable binding of TIRR to any of the DNA substrates tested (Figures 2B and 2C). To confirm these results, we performed electrophoretic mobility shift assays (EMSAs) (Figure S3A) with purified recombinant TIRR (Figure S3B) and radiolabeled ssRNA, RNA beacon, and ssDNA. In agreement with the SPR data, we observed TIRR binding with comparable affinity to the ssRNA and RNA beacon but not to ssDNA. We did not observe any binding of the recombinant 53BP1 Tudor domain to the substrates tested (Figures 2D–2G and S3C–S3F), suggesting that it is unlikely TIRR and 53BP1 would form a complex together with RNA. Taken together, these data show that TIRR binds to ssRNA and RNA hairpin structures.

### Hairpin RNA promotes dissociation of the TIRR/53BP1 complex *in vitro* and *in vivo*

**Figure 3:**
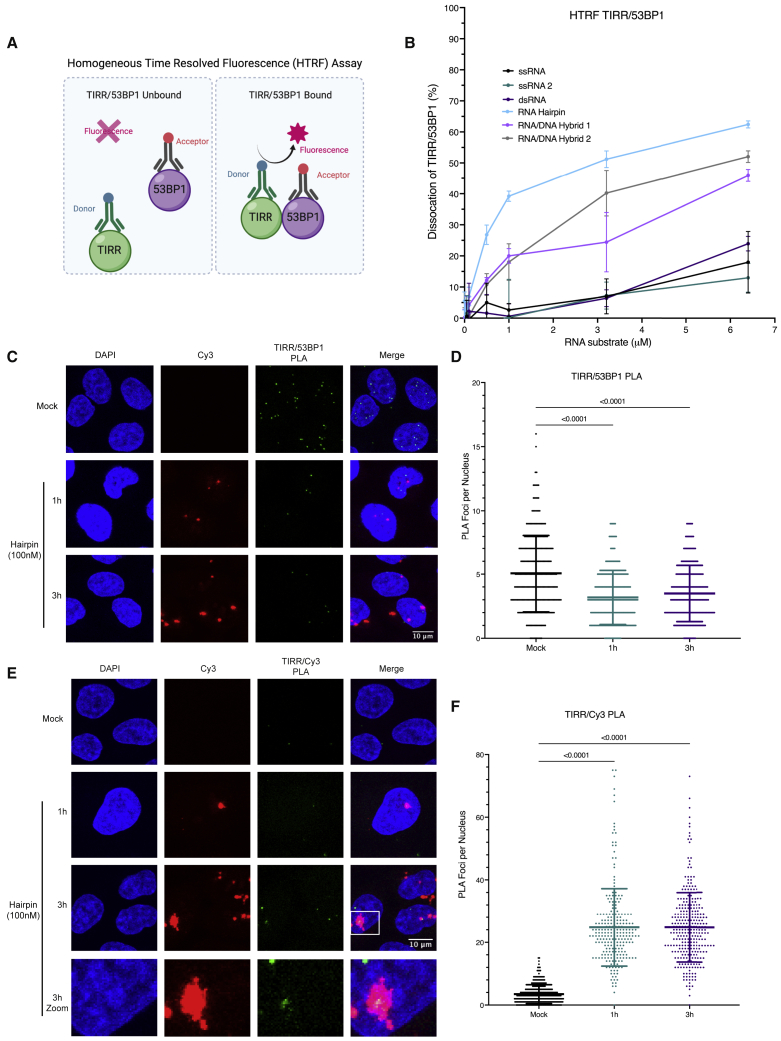
Hairpin RNA promotes dissociation of the TIRR/53BP1 complex *in vitro* and *in vivo* (A) Principles of the homogeneous time-resolved fluorescence (HTRF) assay with TIRR and 53BP1. Image was created with BioRender.com. (B) Graph showing data from the HTRF assay with increasing concentrations of ssRNA 1, ssRNA 2, dsRNA, RNA hairpin, RNA/DNA hybrid 1, and RNA/DNA hybrid 2. (mean ± SD, n = 6). (C) PLA of TIRR and 53BP1 1 or 3 h after transfection of a Cy3-labeled RNA hairpin (100 nM). n(mock) = 275, n(1 h) = 266, n(3 h) = 315. (D) Quantification of (C) (mean ± SD, n = 2). (E) PLA of TIRR and Cy3 1 or 3 h after transfection of a Cy3-labeled RNA hairpin (100 nM). n(mock) = 313, n(1 h) = 241, n(3 h) = 291. (F) Quantification of (E) (mean ± SD, n = 2).

After identifying that TIRR can bind to ssRNA and RNA hairpin structures, we tested if RNA could directly separate the complex of TIRR/53BP1. First, we showed that RNA extracted from the nucleus of irradiated cells was able to reduce binding of 53BP1-Tudor to TIRR in *in vitro* competition assays (Figures S4A–S4C). Next, we performed a homogeneous time-resolved fluorescence (HTRF) assay (Figure 3A) with the different RNA secondary structures in the presence of the TIRR/53BP1 complex. We observed that the RNA hairpin structure was able to separate the TIRR/53BP1 complex (62.4% ± 1.14% dissociation at the highest concentration of RNA tested, mean ± SD), whereas the ssRNA (ssRNA1: 18% ± 9.93%, ssRNA2: 13% ± 4.64%) and dsRNA (24% ± 2.35%) substrates showed only minimal dissociation even at the highest RNA concentrations tested (Figure 3B). To assess if an RNA hairpin could separate TIRR and 53BP1 *in vivo*, we utilized an RNA hairpin that would have no complementarity to other nucleic acids in the cell (Mhlanga et al., 2005) and added a Cy3 label at the 5′ end for visualization. We transfected cells with the Cy3-labeled RNA hairpin structure and confirmed the ability of this hairpin to enter the nucleus, where the TIRR and 53BP1 complex is localized by immunofluorescence (Figure S5A). Next, we performed TIRR and 53BP1 PLA 1 and 3 hours after transfection of 100 nM Cy3-labeled hairpin and observed that transfection of the RNA hairpin was able to reduce the number of PLA foci and, therefore, reduce TIRR and 53BP1 association (Figures 3C and 3D). To check that transfection of the hairpin did not induce DNA damage itself, which could indirectly result in the dissociation of TIRR and 53BP1, we measured the γH2AX levels (phosphorylation of histone H2AX at S139, a marker of DSB induction) by immunofluorescence microscopy and found that the transfection of the hairpin did not induce DSBs itself (Figures S5B and S5C). To assess if this dissociation of TIRR and 53BP1 by the RNA hairpin was a direct result of the interaction of TIRR with the hairpin, we performed PLA with Cy3 and TIRR antibodies, and we were able to observe the interaction of TIRR with the RNA hairpin (Figures 3E and 3F). We also confirmed that an RNA hairpin with a different sequence (Figure S2D) was able to dissociate TIRR and 53BP1, suggesting that this is structure, rather than sequence, specific (Figures S5D and S5E). Together, this suggests that the TIRR/53BP1 dissociation occurred after transfection of the RNA hairpin as a result of the interaction of TIRR with the RNA.

### RNA:DNA hybrids do not show activity on TIRR/53BP1 separation *in vivo*

**Figure 4:**
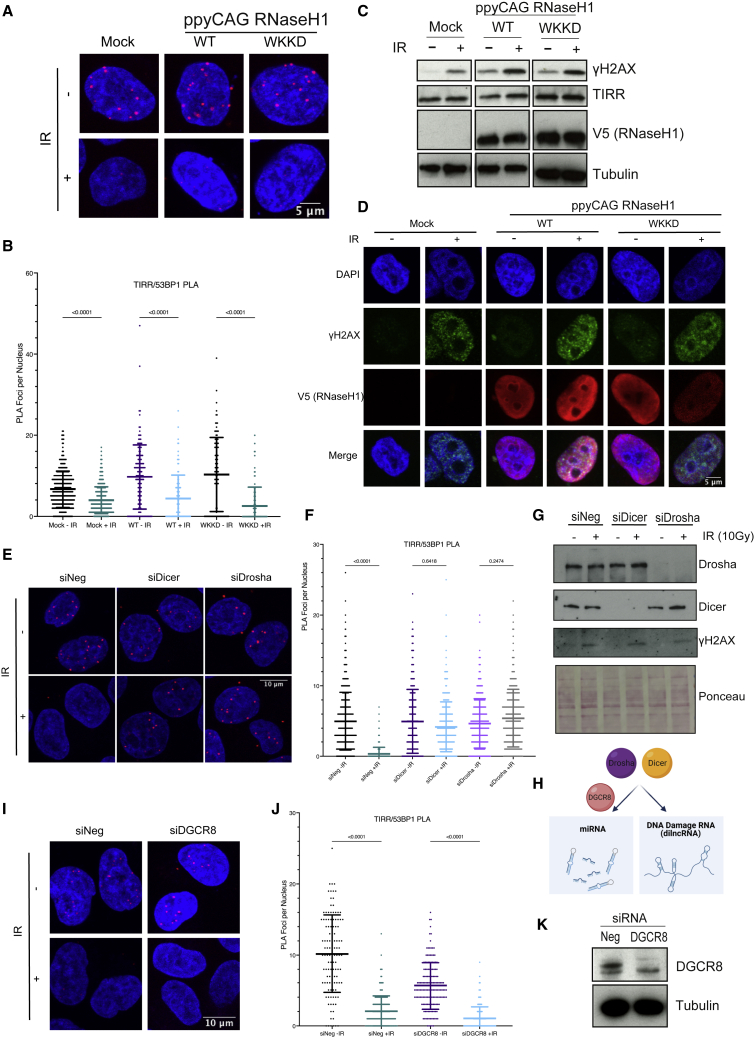
Dicer and Drosha, but not RNA:DNA hybrids, are required for TIRR/53BP1 separation (A) PLA of TIRR and 53BP1 with and without IR (10 Gy, 1 h) with transfection prior to damage of either mock, WT RNasH1, or WKKD mutant RNaseH1. n(mock − IR) = 329, n(mock + IR) = 487, n(WT − IR) = 135, n(WT + IR) = 98, n(WKKD − IR) = 102, n(WKKD + IR) = 89. (B) Quantification of (A) (mean ± SD, n = 2). (C) Western blot showing induction of ɣH2AX after IR and expression of RNaseH1 WT and WKKD mutant. (D) Immunofluorescence microscopy showing induction of ɣH2AX after IR and nuclear localization of RNaseH1. (E) PLA of TIRR and 53BP1 with and without IR (10 Gy, 1 h) with knockdown of Dicer or Drosha prior to damage induction. n(siNeg − IR) = 551, n(siNeg + IR) = 428, n(siDicer − IR) = 459, n(siDicer + IR) = 391, n(siDrosha − IR) = 336, n(siDrosha + IR) = 271. (F) Quantification of (E) (mean ± SD, n = 2). (G) Western blot showing knockdown of Dicer and Drosha and induction of ɣH2AX after IR. (H) Scheme depicting the involvement of Dicer and Drosha in the RNA-dependent DNA-damage response and miRNA pathways, and DGCR8 involvement in miRNA processing. Image was created with BioRender.com. (I) PLA of TIRR and 53BP1 with and without IR (10 Gy, 1 h) with knockdown of DGCR8 prior to damage induction. n(siNeg − IR) = 114, n(siNeg + IR) = 178, n(siDGCR8 − IR) = 155, n(siDGCR8 + IR) = 251. (J) Quantification of (I) (mean ± SD, n = 2). (K) Western blot of DGCR8 knockdown.

RNA:DNA hybrids are known to be associated with DSB repair (Bader and Bushell, 2020; Domingo-Prim et al., 2020; Marnef and Legube, 2021). In the HTRF assay, we saw that RNA:DNA hybrids showed modest TIRR and 53BP1 dissociation activity (RNA:DNA hybrid 1: 46% ± 1.87%, RNA:DNA hybrid 2: 52% ± 1.87%) at the highest RNA concentrations tested (Figure 3B). Therefore, we tested if RNA:DNA hybrids may also impact TIRR/53BP1 dissociation *in vivo* by expressing plasmids encoding wild-type (WT) RNaseH1, which digests the RNA:DNA hybrids, or the binding and catalytic mutant (WKKD) RNaseH1 (Chen et al., 2017). Expression of WT or WKKD RNaseH1 did not impact TIRR/53BP1 dissociation after IR as assessed by PLA with TIRR and 53BP1 antibodies, suggesting that RNA:DNA hybrids do not influence the TIRR/53BP1 complex *in vivo* (Figures 4A and 4B). This is in contrast to what was observed in an *in vitro* setting in HTRF (Figure 3A). This could be due to differences *in vivo*; for example, RNA:DNA hybrids may not be accessible for TIRR and 53BP1 separation in the same way as *in vitro*, such as due to binding of other proteins to RNA:DNA hybrids. Alternatively, this could also potentially be due to a technical issue of RNA:DNA hybrids not being fully removed after RNaseH1 overexpression. Therefore, we tested if RNaseH1 expression in cells was able to actually digest RNA:DNA hybrids by performing immunofluorescence with the S9.6 antibody, which specifically recognizes RNA:DNA hybrids. We observed that the S9.6 signal was reduced after RNaseH1 WT expression, indicating that RNA:DNA hybrids were being digested (Figures S5F and S5G). However, a small amount of S9.6 signal remained after RNaseH1 WT expression. We therefore cannot completely rule out the possibility that the remaining signal may influence TIRR and 53BP1 separation; however, we conclude that RNA:DNA hybrids do not appear to influence TIRR and 53BP1 separation after IR. We also confirmed that γH2AX induction after IR was not affected by RNaseH1 expression and that both WT and WKKD RNaseH1 were nuclear and expressed to similar levels (Figures 4C and 4D).

### Dicer and Drosha, but not DGCR8, are required for TIRR/53BP1 separation

Dicer and Drosha are key components of the miRNA processing pathway and have also been shown to be important in the RNA-dependent DDR (Burger et al., 2017, 2019; Francia et al., 2012). To assess if Dicer and Drosha activity is required for TIRR/53BP1 dissociation, we knocked down Dicer and Drosha using small interfering RNA (siRNA) and found that the complex did not separate after IR in knockdown conditions (Figures 4E–4G). As both Dicer and Drosha are active in the miRNA pathway as well as in the RNA-dependent DDR, we also knocked down DGCR8, a Drosha co-factor involved in miRNA processing but with no known role in the biogenesis or processing of RNA generated at DSBs (Figure 4H). Knockdown of DGCR8 had no impact on TIRR/53BP1 dissociation after IR (Figures 4I–4K), suggesting that the involvement of Dicer and Drosha is independent of their miRNA processing role and likely as a result of their role in the RNA-dependent DDR.

### The TIRR/53BP1 complex is localized to DSBs prior to dissociation

**Figure 5:**
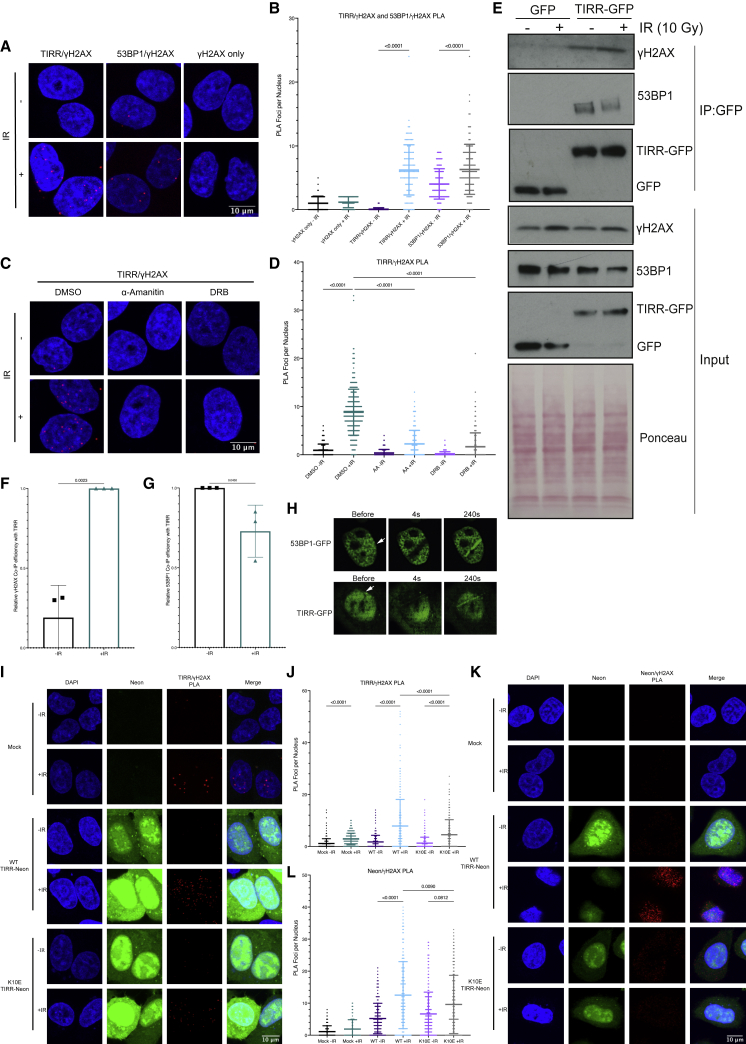
The TIRR/53BP1 complex is localized to DSBs prior to dissociation (A) PLA of TIRR and ɣH2AX, 53BP1 and ɣH2AX, or ɣH2AX alone with and without IR (10 Gy, 1 h) (n(ɣH2AX − IR) = 85, n(ɣH2AX + IR) = 75, n(ɣH2AX + TIRR − IR) = 73, n(ɣH2AX + TIRR + IR) = 105, n(ɣH2AX + 53BP1 − IR) = 123, n(ɣH2AX + 53BP1 + IR) = 122). (B) Quantification of (A) (mean ± SD, n = 2). (C) PLA of TIRR and ɣH2AX with and without IR (10 Gy, 1 h) in the presence of α-Amanitin (2 μg/mL, 24 h) or DRB (100 μM, 1 h). n(DMSO − IR) = 291, n(DMSO + IR) = 357, n(AA − IR) = 303, n(AA + IR) = 303, n(DRB − IR) = 348, n(DRB + IR) = 383. (D) Quantification of (C) (mean ± SD, n = 2). (E) Co-immunoprecipitation of TIRR-GFP or GFP with and without IR (10 Gy, 1 h). Ponceau is used as a loading control. (F) Quantification of (E), ɣH2AX bound to TIRR-GFP ± IR (mean ± SD, n = 3). (G) Quantification of (E), 53BP1 bound to TIRR-GFP ± IR (mean ± SD, n = 3). (H) Laser stripping of 53BP1-GFP U2OS cells and TIRR-GFP HeLa cells (mean ± SD, n = 1). (I) PLA of TIRR and ɣH2AX with and without IR (10 Gy, 1 h) with transfection prior to damage of either mock, WT Neongreen-TIRR, or K10E Neongreen-TIRR. n(mock − IR) = 347, n(mock + IR) = 402, n(WT − IR) = 329, n(WT + IR) = 339, n(K10E − IR) = 432, n(K10E + IR) = 338. (J) Quantification of (I) (mean ± SD, n = 2). (K) PLA of Neongreen and ɣH2AX with and without IR (10 Gy, 1 h) with transfection prior to damage of either mock, WT Neongreen-TIRR, or K10E Neongreen-TIRR. n(mock − IR) = 315, n(mock + IR) = 309, n(WT − IR) = 343, n(WT + IR) = 289, n(K10E − IR) = 324, n(K10E + IR) = 258. (L) Quantification of (K) (mean ± SD, n = 2).

The TIRR/53BP1 complex exists in the nucleoplasm (Drané et al., 2017), but it is not known if its dissociation occurs in the nucleoplasm or if the complex is recruited to the DSB for dissociation to occur. To address this, we tested if TIRR is recruited in proximity to the break itself by performing PLA with TIRR and γH2AX (Rassoolzadeh et al., 2015), and we observed an interaction between TIRR and γH2AX after IR (Figures 5A and 5B). As expected, we also observed an increase in 53BP1 and γH2AX PLA foci after IR, with minimal γH2AX foci visible in the γH2AX-only control (Figures 5A and 5B). Furthermore, the interaction of TIRR with γH2AX was reduced by α-Amanitin and 5, 6-dichloro-1-β-D-ribofuranosylbenzimidazole (DRB) treatments to inhibit RNAPII transcription (Figures 5C and 5D), suggesting that TIRR and 53BP1 recruitment to the DSB sites after damage is RNAPII transcription dependent. The interaction of TIRR with γH2AX after IR was also confirmed by co-immunoprecipitation (coIP) of doxycycline-inducible TIRR-GFP with γH2AX (Figures 5E and 5F). 53BP1 coIP was performed as a control, and as expected, the TIRR and 53BP1 interaction decreased after IR (Figures 5E and 5G). To further explore the recruitment of TIRR to damage sites, we also performed laser striping in live cells expressing TIRR-GFP, or 53BP1-GFP as a control, and detected TIRR-GFP accumulation at the laser stripe (Figures 5H, S6B, and S6C).

Next, we investigated if TIRR and 53BP1 may be recruited to DSBs as a complex for dissociation or if this recruitment of TIRR to damage sites was independent of 53BP1 binding. We performed PLA using plasmids expressing either WT or K10E TIRR, a well-described 53BP1-binding mutant (Botuyan et al., 2018; Dai et al., 2018; Wang et al., 2018), tagged with Neongreen. Both the WT and K10E Neongreen-TIRR were expressed to comparable levels (Figure S6A). PLA using a combination of either γH2AX and TIRR antibodies (Figures 5I and 5J), or γH2AX and Neongreen antibodies (Figures 5K and 5L), revealed that K10E TIRR proximity to γH2AX was reduced compared with WT TIRR. This suggests that the recruitment of TIRR, which cannot bind 53BP1, to DSB sites is impaired, and TIRR and 53BP1 may therefore be recruited to the DSB site as a complex for dissociation in proximity to the break.

Furthermore, performing laser striping on cells transiently transfected with WT or K10E Neongreen-TIRR revealed that K10E TIRR accumulation at the stripe is impaired relative to the WT TIRR (Figures S6D and S6E), in agreement with the PLA data. Additionally, the recruitment of both TIRR-GFP and 53BP1-GFP to the stripe was also impaired when cells were treated with DRB prior to the laser-induced damage (Figures S6F–S6I). To evaluate the contribution of RNAPII directly or RNA transcribed by RNAPII, we performed coIP on TIRR-GFP and assessed for RNAPII binding. We were unable to observe any binding of RNAPII to TIRR in this assay (Figure S6J), suggesting that the recruitment of TIRR to DSBs is more likely dependent on RNAPII transcription rather than RNAPII binding. Taken together, these data suggest that TIRR and 53BP1 are recruited to DSBs likely as a complex, and this is dependent on RNAPII transcription.

### The TIRR/53BP1 complex is in close proximity to RNA transcribed at the DSB

**Figure 6:**
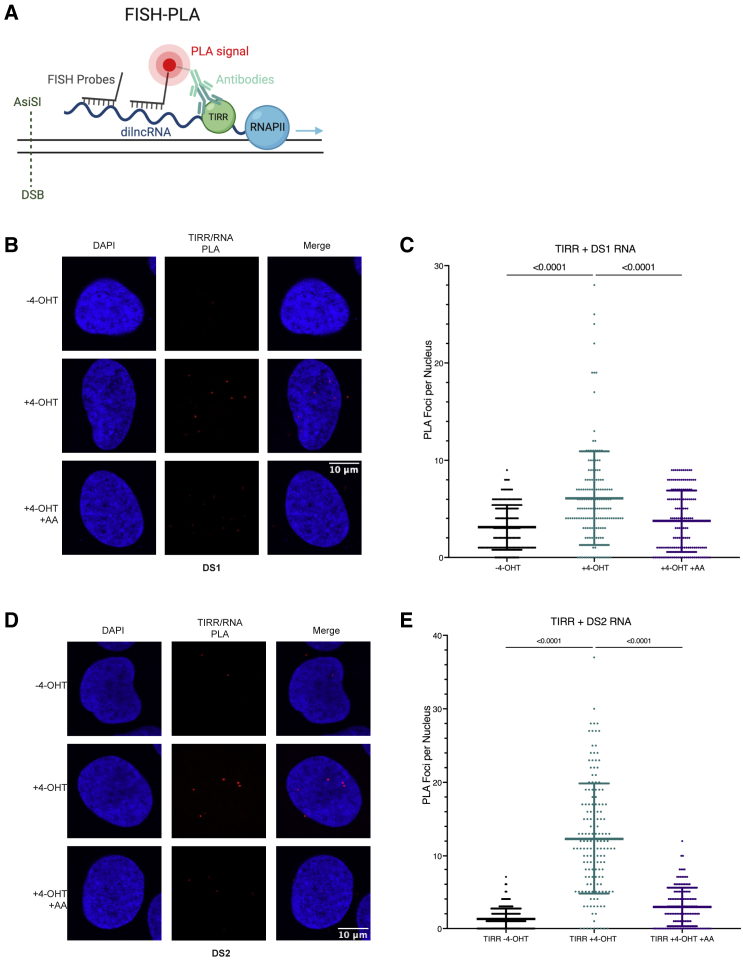
The TIRR/53BP1 complex is in close proximity to RNA transcribed at the DSB (A) Representation of the FISH-PLA assay. Image was created with BioRender.com. (B) FISH-PLA of TIRR and DS1 probes, with and without 4-OHT (500 nM, 4 h) or α-Amanitin (2 μg/mL, 24 h). n(− 4-OHT) = 179, n(+ 4-OHT) = 154, n(− 4-OHT + AA) = 143. (C) Quantification of (B) (mean ± SD, n = 2). (D) FISH-PLA of TIRR and DS2 probes, with and without 4-OHT (500 nM, 4 h) or α-Amanitin (2 μg/mL, 24 h). n(− 4-OHT) = 177, n(+ 4-OHT) = 155, n(− 4-OHT + AA) = 136. (E) Quantification of (D) (mean ± SD, n = 2).

Our data suggested that damage-induced RNA transcripts transcribed *de novo* from the break itself by RNAPII may be responsible for dissociation of TIRR and 53BP1 at DSBs. To determine if DSB-derived RNA was directly involved in TIRR/53BP1 dissociation, we designed a sequence-specific assay; FISH-PLA. For FISH-PLA, we employ the U20S-*Asi*SI-ER cell line, where the *Asi*SI restriction enzyme is joined with a modified ligand-binding domain of the estrogen receptor (ER), which translocates to the nucleus after addition of 4-hydroxytamoxifen (4-OHT), after which *Asi*SI cuts in a sequence-dependent manner to induce site-specific DSBs (Massip et al., 2010). After incubation with 500 nM 4-OHT for 4 h, we can observe a significant increase in γH2AX foci, indicative of induction of sequence-specific DSBs by the *Asi*SI enzyme. The number of γH2AX foci was also not altered by the addition of α-Amanitin prior to 4-OHT addition (Figures S7A and S7B). Using this system, we designed probes complementary to the RNA transcript that would be transcribed at specific cut sites (plus probes), which contain an adaptor sequence at the ends that can ligate with a PLA minus probe (Figure 6A). PLA with TIRR and the probes for RNA from cut site DS1 (located in the promoter region of RBMXL1/CCBL2 [Figure S7C]) revealed the proximity of TIRR with RNA produced at the break after 4-OHT incubation, which was reduced by α-Amanitin treatment prior to 4-OHT addition (Figures 6B and 6C). This same result was also obtained with probes designed to a different cut site, DS2 (located in the genic region of VSTM2B [Figure S7D]) (Figures 6D and 6E). We also performed FISH-PLA using an antibody against 53BP1 with the DNA probes and saw that similarly to TIRR, 53BP1 was also in close proximity to RNA produced at the break at both DS1 (Figures S7E and S7F) and DS2 (Figures S7G and S7H). This suggests that TIRR/53BP1 are in proximity to RNA originating from the break itself.

### Hairpin RNA binding to TIRR is incompatible with 53BP1 binding

**Figure 7:**
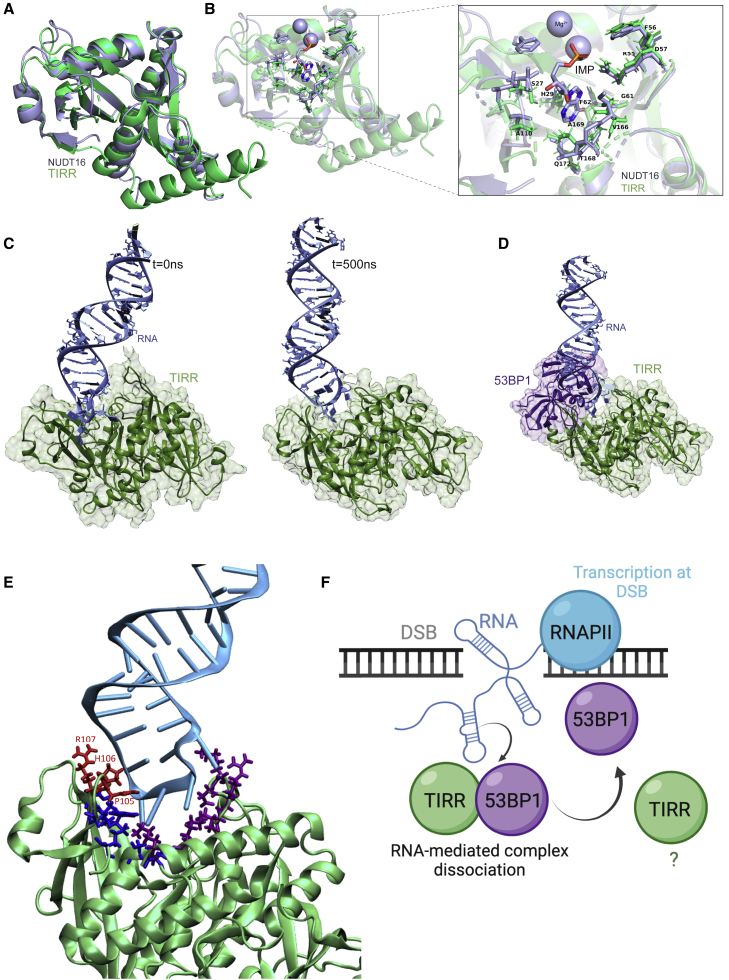
Hairpin RNA binding to TIRR is incompatible with 53BP1 binding (A) Overlay of NUDT16 (PDB: 3COU) and TIRR (PDB: 6D0L). (B) The residues on NUDT16 within 5 Å of IMP (PDB: 2XSQ) and the corresponding residues on TIRR (PDB: 6D0L) are highlighted. (C) Complex of the TIRR dimer (green) with the RNA hairpin (cyan) at the start (after equilibration, t = 0 ns, left panel) and after 500 ns of MD simulation (right panel). (D) Complex of the TIRR protein (green) with the RNA hairpin (cyan) after 500 ns of MD simulation overlapped with the TIRR/53BP1 (purple) complex. (E) Complex of TIRR (green) with the RNA hairpin (cyan) with highlighted residues (liquorice) in TIRR interacting with the RNA. In red are the residues known to interact and recognize 53BP1, in blue are additional residues of the TIRR monomer interacting with the RNA, and in purple are residues of the TIRR dimer interacting with the RNA. (F) Model of RNA-mediated dissociation of TIRR and 53BP1 in response to DSBs. Image was created with BioRender.com.

Finally, to understand how RNA could impact the TIRR/53BP1 interaction, we modeled TIRR bound to the hairpin RNA. The RNA-binding site on TIRR has not been fully characterized, so we utilized the structural similarity of TIRR with its paralog NUDT16 (Figure 7A), for which a crystal structure exists bound to a nucleic acid substrate IMP, to set active residues on TIRR for modeling (Figure 7B). This site overlaps with the site identified by RNA crosslinking mass spectrometry (He et al., 2016) and the 53BP1 binding site. The model generated using HADDOCK was then used to run molecular dynamics (MD) simulations after adjusting the protein-RNA interaction (see STAR Methods) into the known RNA-binding cavity (Botuyan et al., 2018). We ran the MD simulation of the TIRR dimer (the physiological state of TIRR) in complex with the RNA hairpin (see STAR Methods), and we observed that during the simulation, the RNA moves and adapts to the protein cavity to improve the interactions with TIRR (Figure 7C). We then checked if the key residues in the interaction between 53BP1 and TIRR were also important for the interaction of TIRR with the RNA hairpin. Overlapping the TIRR/53BP1 complex, with the RNA hairpin after 500 ns of simulation shows that the hairpin and the 53BP1 occupy almost the same site (Figure 7D), and many residues are maintained in the interaction with the hairpin (residues marked in red in Figure 7E). During the simulation, other residues improved and stabilized the interactions between the RNA and TIRR dimer. Among those residues, we found mainly arginines and lysines but also serines, valines, and phenylalanine (the closest residues at the end of the simulation are highlighted in blue in Figure 7E). We detected an improvement in the interaction energy given by the contacts between the second protein of the TIRR dimer and the RNA hairpin (mainly lysines and arginines; in purple in Figure 7E). We did the same analysis using the monomer of TIRR, and we noticed that during the simulation, the RNA hairpin, initially placed by HADDOCK (see STAR Methods) in the protein cavity, moves and, lacking the interaction with the second protein of the dimer, has a bigger displacement (Figures S8A and S8B). Also, in this case, the RNA occupies the same site as 53BP1 (Figures S8C and S8D), and key residues on TIRR involved in the interaction with 53BP1 are also involved in the interaction with the hairpin (in red in Figure S8E). Together, these simulations highlight that the binding of RNA to TIRR is incompatible with 53BP1 binding and suggest that RNA may displace and compete with 53BP1 through occupation of the 53BP1 binding site on TIRR.

## Discussion

Collectively, our data suggest that DNA-damage-induced RNA produced at DSBs by RNAPII transcription modulates repair of DSBs by facilitating the dissociation of TIRR and 53BP1 (Figure 7F). Unexpectedly, it appears that the TIRR and 53BP1 complex is brought in close proximity to the DSB as a complex, which then allows the RNA transcribed from the break to separate the complex. After dissociation, 53BP1 is then free to bind the chromatin through its Tudor domain, although the destination of TIRR after dissociation remains unknown. We found that the structure of the RNA plays an important role in separation of TIRR and 53BP1, with a hairpin structure able to dissociate TIRR and 53BP1 both *in vitro* and *in vivo*. TIRR can bind to ssRNA and hairpin RNA, with a weak approximate affinity (Kd) of ∼100 μM. The binding affinity of 53BP1 to TIRR has been shown to be around 1 μM (Dai et al., 2018), so a direct competition of the hairpin RNA alone with 53BP1 for TIRR binding would likely be inefficient *in vivo*. One explanation could be that TIRR itself precipitates *in vitro* at low salt concentrations needed to foster RNA-protein interactions, and it is possible that the binding affinity for TIRR with the RNA is underestimated as a result. Additionally, RNA modifications (Jimeno et al., 2021), such as m^6^A (Zhang et al., 2020) and m^5^C (Chen et al., 2020), play important roles in DSB repair, and it is possible that the RNA responsible for TIRR/53BP1 dissociation could be modified. Furthermore, other proteins or post-translational modifications may contribute to this process of dissociation, such as ATM phosphorylation of 53BP1 (Botuyan et al., 2018; Drané et al., 2017). In agreement with this literature, we found that ATM inhibition impaired the dissociation of TIRR and 53BP1 after IR (Figures S9A–S9C); however, RNAPII inhibition using either α-Amanitin or DRB, and Dicer and Drosha knockdown, did not influence pATM (S1981) after IR (Figure S9D). This suggests that the RNA-dependent DDR may contribute to TIRR/53BP1 dissociation in concert with other pathways, such as ATM phosphorylation of 53BP1. Nevertheless, we provide proof of principle that RNA can dissociate the TIRR/53BP1 complex and that RNA transcripts from DSBs themselves are the potential source of this RNA. Given that the FISH-PLA probes are designed to a specific genomic locus, we would expect to see only 1 or 2 FISH-PLA foci after 4-OHT addition. We see an average of 5 PLA foci for 53BP1 after +4-OHT and an average of 6 PLA foci for TIRR at DS1, with some cells containing as many as 10 foci or more. This could suggest a potential interaction with DSB-derived RNA processed from the longer dlincRNA/DART transcript at the break, as the 11 individual probes span a region up to ∼1.2 kb away from the cut site. Additionally, it is not yet known exactly what structure (or structures) RNA transcribed at DSBs forms, although RNA:DNA hybrids as well as dsRNA have been described, likely aided by the availability of experimental tools such as antibodies recognizing these structures. However, we do know that transcription at DSBs can extend up to 1–3 kb away from the break end (Bonath et al., 2018; Burger et al., 2019; Michelini et al., 2017). We hypothesize that given the length of these transcripts, the dilncRNA is able to form RNA secondary structures, such as RNA hairpin-like structures, which could influence TIRR and 53BP1 separation.

MD simulations of TIRR in complex with hairpin RNA highlight how RNA impairs the interaction of TIRR and 53BP1, with the hairpin RNA binding to TIRR being incompatible with 53BP1 binding to TIRR. Many of the same residues that are important in TIRR interaction with 53BP1 (such as Arg107) are important in the modeled interaction of TIRR with the RNA. Together, these data suggest that RNA can directly compete for TIRR interaction with 53BP1, and there is considerable overlap between residues involved in RNA interaction and 53BP1 interaction on TIRR.

Understanding how TIRR influences 53BP1 function is crucial for understanding DNA repair and the mechanisms of cancer progression. Interestingly, we also found that TIRR and 53BP1 can be dissociated by different drugs, which can lead to the production of DNA DSBs as part of their mechanism of action, including cisplatin (Figures S10A and S8B) and olaparib (Figures S10C and S10D) and not only IR and Eto. This implies that regardless of how the DSB is induced, TIRR and 53BP1 will dissociate, which has interesting implications in anti-cancer treatment. TIRR itself, found in the chromosomal region 16p13.3, is also frequently amplified in cancer, in particular breast cancers (Drané et al., 2017). It is interesting to note that when comparing TIRR amplification with any amplifications of genes in the 16p13.3 region, TIRR is always amplified as part of a larger 16p13.3 region (Figure S10E). This chromosomal region is amplified in a selection of different cancers (Figure S10F), although TIRR amplification within this 16p13.3 region is predominantly amplified in breast cancer (Figure S10F). Interestingly, TIRR amplification is associated with increased TIRR RNA expression (Figure S10G), which correlates with TIRR protein expression in breast cancers (Figure S10H). Increased TIRR expression is associated with deregulation of 53BP1 function (Botuyan et al., 2018; Drané et al., 2017; Wang et al., 2018) and has been suggested as a potential mechanism of PARPi-resistance development. Therefore, understanding how the TIRR and 53BP1 complex is regulated, including by RNA, could help us target this complex and enhance the sensitivity of HR-deficient cancer cells to PARPis.

### Limitations of the study

Currently, it is not clear if DSBs occurring in different regions of the genome, such as transcribed or intergenic regions, would result in a different outcome for the separation of TIRR and 53BP1, raising the question; do all DSBs result in RNA-mediated separation of TIRR and 53BP1? *De novo* transcription has been observed at DSBs occurring at different regions of the genome, including genic and intergenic regions (D’Alessandro et al., 2018; Michelini et al., 2017), although not unequivocally (Burger et al., 2019; Domingo-Prim et al., 2019; Vítor et al., 2019). Therefore, it is possible that TIRR/53BP1 separation may be regulated differently depending on where in the genome DSBs occur. The resolution of PLA does not allow us to look at the separation of TIRR/53BP1 when DSBs are induced at a single site, which would allow the assessment of TIRR/53BP1 separation when DSBs are induced at different regions of the genome; however, this would be an interesting focus of future studies.

It is also not clear exactly what the RNA that can separate TIRR and 53BP1 looks like. We find that an RNA hairpin secondary structure has the capacity to separate TIRR and 53BP1 *in vitro* and *in vivo*; however, it is certainly possible that the actual RNA structure produced at breaks may differ slightly from this. We hypothesize that dilncRNA/DARTs, which can be transcribed up to 1–3 kb away from the break, can form secondary structures, such as hairpins, which we believe could separate TIRR and 53BP1. We also did not find a role of RNA:DNA hybrids in this study, although we cannot completely rule this out, as RNaseH1 overexpression may not be 100% efficient in degrading all RNA:DNA hybrid structures, as assessed by S9.6 immunofluorescence. Additionally, RNA modifications have recently been implicated in DNA repair (Jimeno et al., 2021), and as such, it would be interesting to assess the contribution of RNA modifications to TIRR and 53BP1 separation in future studies. Furthermore, how other RNAs in the cell, such as lncRNA and miRNA, in a normal non-damage condition do not influence TIRR and 53BP1 dissociation is an open question. We speculate that this could be due to other damage-responsive signaling, such as RNA modification or ATM signaling. Moreover, how RNA-mediated separation is coordinated with other signaling pathways, such as ATM signaling, is currently unclear. It is possible that RNA-mediated separation occurs in conjunction with other signaling pathways, or as separate redundant pathways, or potentially differentially regulating TIRR and 53BP1 separation when DSBs occur in different genomic regions. These questions should be answered in future studies.

## STAR★Methods

### Key resources table

**Table:** 

REAGENT or RESOURCE	SOURCE	IDENTIFIER
**Antibodies**		

DGCR8 C-terminal Polyclonal antibody	Proteintech	10996-1-AP; RRID:AB_2090987
Drosha (D28B1) Rabbit mAb	Cell Signaling Technology	3364S; RRID:AB_2238644
Anti-Dicer antibody [13D6]	Abcam	ab14601; RRID:AB_443067
53BP1 Antibody	Novus Biologicals	NB100-304; RRID:AB_10003037
Purified Mouse Anti-Human 53BP1	BD Transduction Laboratories	612522; RRID:AB_2206766
Anti-53BP1 Antibody, clone BP13	Sigma	MAB3802; RRID:AB_11212586
NUDT16L1 Antibody	Novus Biologicals	NBP1-92209; RRID:AB_11024857
Polyclonal Anti-NUDT16L1 Antibody	Atlas Antibodies	HPA044186; RRID:AB_10968571
Anti-gamma H2A.X (phospho S139) antibody	Abcam	ab11174; RRID:AB_297813
Anti-beta Tubulin antibody - Loading Control	Abcam	ab6046; RRID:AB_2210370
mNeonGreen antibody	Proteintech	32f6; RRID:AB_2827566
Anti-phospho-Histone H2A.X (Ser139) Antibody, clone JBW301	Sigma	05–636; RRID:AB_309864
Anti-anti-Cy3 Antibody (A-6)	Santa Cruz	sc-166894; RRID:AB_10614143
Anti-RNA polymerase II CTD repeat YSPTSPS antibody	Abcam	ab26721; RRID:AB_777726
GFP Monoclonal antibody	Chromotek	3h9; RRID:AB_10773374
Anti-V5 tag antibody [SV5-P-K]	Abcam	ab206566; RRID:AB_2819156
Anti-DNA-RNA Hybrid Antibody, clone S9.6	Merck	MABE1095; RRID:AB_2861387

**Bacterial and virus strains**		

BL21(DE3) Competent *E. coli*	New England BioLabs	C2527H
Rosetta™(DE3) Competent Cells	Novagen	70954

**Chemicals, peptides, and recombinant proteins**		

TP53BP1 tudor-like region (human recombinant)	Cayman	14073-100ug-CAY
α-amanitin	Cayman	17898
Etoposide	Cayman	12092
DRB	Cayman	10010302
Doxycycline	Cayman	14422-5 g-CAY
ML-60218	Millipore	557403
Cisplatin	Sigma	P4394
Olaparib	Cayman	10621-10mg-CAY
4-OHT	Cayman	14854-1mg-CAY
KU-60019	Tocris	4176

**Critical commercial assays**		

Duolink® In Situ Red Starter Kit Mouse/Rabbit	Sigma	DUO92101-1KT
Monarch Total RNA Miniprep Kit	New England BioLabs	NEB #T2010

**Deposited data**		

BIGNASIM	N/A	N/A

**Experimental models: Cell lines**		

Hela	ATCC	CCL-2
TRex Flp-IN shTIRR Hela	A gift from Rosario Avolio	N/A
TRex Flp-IN shGFP Hela	A gift from Rosario Avolio	N/A
TRex Flp-IN TIRR-GFP Hela	A gift from Rosario Avolio	N/A
TRex Flp-IN GFP Hela	A gift from Rosario Avolio	N/A
53BP1-GFP U2OS	A gift from Matthias Altmeyer	N/A
A*si*SI-ER U2OS	A gift from Gaëlle Legube	N/A

**Oligonucleotides**		

ON-TARGETplus Human DGCR8 siRNA	Dharmacon	L-015713-00-0005
ON-TARGETplus Human DROSHA siRNA	Dharmacon	L-016996-00-0005
ON-TARGETplus Human DICER1 siRNA	Dharmacon	L-003483-00-0005
ON-TARGETplus Non-targeting Control	Dharmacon	D-001810-01-20

**Recombinant DNA**		

ppyCAG_RNaseH1_WT	Addgene	#111905
ppyCAG_RNaseH1_WKKD	Addgene	#111906

**Software and algorithms**		

GraphPad Prism 9	GraphPad Software, San Diego, California USA, www.graphpad.com	N/A
HADDOCK2.4	van Zundert and Bonvin (2014)	N/A
PyMOL	Schrodinger LLC (2021)	N/A
AMBER 20	Case et al. (2021)	N/A
BIGNASIM	Hospital et al. (2016)	N/A
CPPTRAJ	Roe and Cheatham (2013)	N/A
VMD v1.9.4	Humphrey et al. (1996)	N/A
RNAfold	Lorenz et al. (2011)	N/A
RNAComposer	Biesiada et al. (2016)	N/A
Fiji	Schindelin et al. (2012)	N/A
CellProfiler	Carpenter et al. (2006)	N/A
BioRender	https://biorender.com/	N/A

### Resource availability

#### Lead contact

Further information and requests for resources and reagents should be directed to and will be fulfilled by the Lead Contact, Monika Gullerova (monika.gullerova@path.ox.ac.uk).

#### Materials availability

Reagents generated in this study can be made available on request.

### Experimental model and subject details

#### Cell lines

HeLa Wild Type, TRex Flp-IN shTIRR HeLa, TRex Flp-IN shGFP HeLa, TRex Flp-IN TIRR-GFP HeLa, TRex Flp-IN GFP HeLa (all Flp-IN cell lines were a gift from Rosario Avolio), 53BP1-GFP U2OS (a gift from Matthias Altmeyer), and *Asi*SI-ER U2OS cells (a gift from Gaëlle Legube) were cultured in DMEM with 1% L-glutamine, and 10% FBS, with and without antibiotics in 5% CO_2_ at 37°C.

Ionising Radiation induced damage was delivered at the indicated doses using the Gravatom (GRAVITRON RX 30/55).

### Method details

#### RNA and DNA transfections

Short interfering RNA (siRNA) for gene expression knockdown was performed using Lipofectamine RNAimax according to the manufacturer’s instructions. Cells were seeded in antibiotic free media, and forward transfected 24 h later with 20nM siRNA and incubated for 48 h unless otherwise stated. siRNA used; ON-TARGETplus Non-targeting Control (Dharmacon), ON-TARGETplus Human DGCR8 siRNA (Dharmacon), ON-TARGETplus Human DICER1 siRNA (Dharmacon), ON-TARGETplus Human DROSHA siRNA (Dharmacon).

For transfection of the Cy3 labeled RNA hairpin, the RNA was annealed at a concentration of 10μM in 1X annealing buffer (50mM Tris pH7.5, 50mM NaCl) by heating at 98°C for 3 min, followed by slowly cooling to room temperature. RNA was transfected using Lipofectamine RNAimax with either 20nM (low) or 100nM (high) RNA concentrations.

Plasmids were transfected using Lipofectamine 3000 according to the manufacturer’s instructions. Cells were seeded and 24 h later forward transfected with 1-5μg of plasmid DNA. Cells were harvested 24–48 h later. Plasmids ppyCAG_RNaseH1_WT and ppyCAG_RNaseH1_WKKD were a gift from Xiang-Dong Fu (Addgene plasmid #111905 and #111906).

#### RNA and DNA sequences

RNA and DNA oligonucleotides were purchased from either IDT or Sigma. RNA sense (5′ → 3′): GGA CAA GAU GCC UUA UGU GG; RNA Antisense: CCU GUU CUA CGG AAU ACA CC; RNA Hairpin: GGA CAA GAU GCC UUA UGU GG ACU UCA CCA CAU AAG GCA UCU UGU CC; RNA Beacon: CGC UCC CAA AAA AAA AAA CCG AGC G; DNA sense: GGA CAA GAT GCC TTA TGT GG; DNA Antisense: CCT GTT CTA CGG AAT ACA CC; DNA Hairpin: GGA CAA GAT GCC TTA TGT GG ACT TCA CCA CAT AAG GCA TCT TGT CC; Cy3 Labeled RNA Hairpin: Cy3 GCC GGG CGA AGA AUG AAA ACG GC.

#### Western Blotting

Protein samples in 1X Laemmli Buffer (Alfa Aesar) were sonicated for 10–30 s, followed by incubation for 5 min at 98°C. Samples were then loaded in 4-15% Mini-Protean TGX gels (Biorad) and electrophoresed in Tris-Glycine running buffer (Biorad) at 100V for 1 h. Gels were transferred onto nitrocellulose membranes for 1 h 30 min at a constant voltage of 15V. Successful transfer of protein to the membrane was visualised using Ponceau S (Merck). Membranes were blocked in 5% milk or BSA in PBS with 0.1% Triton X-100 (PBST) at room temperature for 1 h. Membranes were then incubated with primary antibodies overnight at 4°C. Membranes were washed 3 times in PBST and then incubated with a secondary antibody for 1 h at room temperature. Membranes were washed 3 times in PBST, then visualised using ECL and Amersham Hyperfilm ECL film. Some membranes were processed using the Super Signal Western Blot Enhancer kit (Thermo Fisher) according to the manufacturer’s instructions.

#### Immunofluorescence (IF)

Cells were plated on glass cover slips. Cover slips were washed 3 times in cold PBS and fixed in cold 4% paraformaldehyde (Alfa Aesar) in PBS for 10 min. Cover slips were then washed 3 times in cold PBS and permeabilised with cold 0.2% Triton X-100 in PBS (PBST) for 10 min. Slides were then washed 3 times in cold PBS and blocked in 10% FBS in PBS for 2 h at 4°C. Primary antibodies were diluted to the appropriate concentration in 10% FBS in PBS and incubated overnight at 4°C in a humidified chamber. Cover slips were then washed 3 times in PBST. Coverslips were incubated for 2 h at RT with secondary antibodies diluted in 0.15% FBS in PBS. Coverslips were then washed 3 times in PBST in the dark. Coverslips were then washed a final time in PBS before mounting on slides with Fluoroshield Mounting Medium with DAPI (Abcam). Slides were imaged using an Olympus Fluoview FV1200 confocal microscope with a 60X objective lens.

For S9.6 Immunofluorescence experiments, after fixation permeabilisation with 0.2% Triton X-100, cells were treated with RNase pre-treatment solution (0.1% BSA, 3mM MgCl_2_, RNAse T1 (Thermo) 1:200 (v/v), ShortCut RNAseIII (NEB) 1:200 (v/v) in PBS) for 2 h at RT (Alagia et al., 2022). Subsequent blocking and antibody incubation steps were then carried out as above.

#### Proximity Ligation Assay (PLA)

Proximity Ligation Assay was performed using the Duolink In Situ Red or Green Starter Kit Mouse/Rabbit (Sigma) according to the manufacturer’s instructions. Briefly, cells were seeded on coverslips, fixed and permeabilized according to the immunofluorescence protocol. Cover slips were blocked in PLA blocking buffer in a humidity chamber for 1 h at 37°C. Primary antibodies were diluted in PLA antibody diluent and incubated with coverslips in a humidified chamber at 4°C overnight. Cover slips were then washed 3 times in PLA buffer A at RT, then incubated with PLA probes for 1 h at 37°C. Cover slips were washed 3 times in PLA buffer A, then incubated with PLA ligase in ligation buffer for 30 min at 37°C. Coverslips were washed 3 times in PLA buffer A and incubated with PLA polymerase in polymerase buffer for 100 min at 37°C in the dark. Cover slips were washed 3 times in PLA buffer B in the dark, and twice in 0.01X PLA buffer B. Coverslips were mounted on slides using PLA DAPI solution and imaged using an Olympus Fluoview FV1200 confocal microscope with a 60X objective. Images were visualised using Fiji (Schindelin et al., 2012) and analyzed using CellProfiler (Carpenter et al., 2006) with the Speckle Counter pipeline.

#### Co-Immunoprecipitation (Co-IP)

Cells were harvested from in 10cm dishes by washing 3x in cold PBS, followed by scrapping in PBS, and centrifuged at 500g for 5 min. The cell pellet was lysed in 5 volumes Co-IP lysis buffer (50mM Tris pH 8, 150mM NaCl, 1mM EDTA, 5mM MgCl_2_, 0.5% NP40, Pierce Universal Nuclease for Cell Lysis (Thermo Fisher), Protease inhibitors and Phosphatase inhibitors (Roche)) for 30 min at 4°C on a wheel. Lysates were centrifuged at 17,000g for 10 min at 4°C. 1.5x volume of Co-IP dilution buffer (50mM Tris pH 8, 150mM NaCl, 1mM EDTA, 5mM MgCl_2_, Protease inhibitors and Phosphatase inhibitors (Roche)) was added to the supernatant. GFP-Trap magnetic beads (Chromotek) were washed 3x in cold dilution buffer and added to the lysate on a wheel at 4°C for 1.5 h. The beads were then washed 3x in cold Co-IP dilution buffer and samples were eluted using 2x Laemmli buffer (Alfa Aesar) by heating at 95°C for 10 min.

#### RNA extraction

Total or nuclear RNA was extracted using either TRIzol (Life Technologies) or RNA lysis reagent from the Monarch Total RNA Miniprep Kit (NEB) as per the manufacturer’s instructions. For TRIzol extraction, TRIzol was added to cells, vortexed, and chloroform was added to the TRIzol sample according to the manufacturer’s instructions (200μL of chloroform per 1mL of TRIzol Reagent) and vortexed, before centrifugation at 12,000g for 15 min at 4°C. The aqueous phase was collected and used in the Monarch Total RNA Miniprep Kit (NEB) as per the manufacturer’s instructions. RNA was eluted in water and quantified using the Implen Nanophotometer N60.

#### Live cell striping

Cells were seeded in 35 mm glass bottom dishes. Before laser stripping, cells were pre-sensitised using Hoechst 33342 (10μg/mL) at 37°C in phenol red free media. Cells were imaged using an Olympus SoRa spinning disc confocal microscope using the 405nm FRAP module in a chamber at 37°C and 5% CO_2_. To select for cells with similar expression levels, cells were systematically chosen using a 488-nm laser setting. DNA damage was induced by a 405nm laser and recruitment of the fluorescently labeled proteins was monitored by live cell imaging. All images were quantified using FIJI software. Fluorescent intensities were normalised against intensities measured in undamaged parts of nuclei.

#### *In vitro* RNA separation assays

To perform *in vitro* RNA separation assays for TIRR and 53BP1 with increasing concentrations of nuclear irradiated RNA, 1μg of recombinant Flag-TIRR and 1μg recombinant HA-53BP1Tudor were incubated together with 20μL of Anti-FLAG M2 magnetic beads (Sigma) per sample in TGEN150 buffer (20mM Tris pH 7.65, 150mM NaCl, 3mM MgCl_2_, 10% Glycerol, 0.01% NP40, Protease inhibitors (Roche)) for 1 h at 4°C on a wheel. The supernatant was removed and the beads washed 1X in TGEN150 buffer. Nuclear RNA from HeLa cells 1h post 10Gy IR was extracted using TRIzol followed by Monarch Total RNA Miniprep Kit. Increasing amounts of RNA (500ng, 1μg, 5μg, 10μg) was added to the beads in constant volume of TGEN150 buffer with Ribolock (Life Technologies) and incubated at RT on a wheel for 1 h. Beads were washed 2X in TGEN150 buffer, before being eluted in 2X Laemmli SDS sample buffer (Alfa Aesar) by boiling at 95°C for 10 min. Eluant was collected and ran on a Western blot to visualise binding.

#### FISH-PLA

A*si*SI U2OS Cells were plated on glass coverslips and 24 h later incubated with α-amanitin (2μg/mL) where indicated. 24 h after addition of α-amanitin, Tamoxifen (4-OHT) was added at a concentration of 500nM where indicated for 4 h. Coverslips were then washed 3 times in cold PBS and fixed in cold 4% paraformaldehyde (Alfa Aesar) in PBS for 15 min. Coverslips were then washed 3 times in cold PBS and permeabilised with cold 0.5% Triton X-100 in PBS (PBST) for 10 min. Slides were then washed 3 times in cold PBS and blocked in blocking buffer (1x SSC, 20μg/μL yeast tRNA (Life Technologies), 20μg/μL salmon sperm DNA (Life Technologies), 0.5% Triton X-100, 2% BSA, 1U/μL RNAsin Plus (Promega)) at 37°C for 1 h. Probe incubation buffer (1x SSC, 100nM DNA probes) was heated to 98°C for 3 min and cooled on ice for 10 min before addition of 1% Triton X-100 and 1U/μL RNAsin Plus (final). Coverslips were incubated with probe incubation buffer for 3 min at 80°C, and then at 37°C overnight. Coverslips were washed 3x in SSC-T buffer (1x SSC, 0.1% Triton X-100, 1% BSA) and 2x in PBS, before blocking for 1 h at 37°C with PLA blocking buffer. Primary antibodies were diluted to the appropriate concentration in PLA antibody diluent and incubated overnight at 4°C in a humidified chamber. Cover slips were then washed 3 times in PLA buffer A at RT, then incubated with minus rabbit PLA probe (Sigma) for 1 h at 37°C. The PLA protocol was then followed according to the manufacturer’s instructions using the Duolink In Situ Red Starter Kit Mouse/Rabbit (Sigma).

DS1: RBMXL1 (promoter) hg19, chr1:89,458,643

DS2: VSTM2B (CDS) hg19, chr19:30,019,487

Sequences of FISH-PLA Probes can be found in supplemental information (Table S1).

#### Protein purification for EMSA experiments

Rosetta™(DE3) E. coli cells from a glycerol stock were grown in TB supplemented with 8g/L glycerol, 100μg/mL kanamycin and 34μg/mL chloramphenicol at 37°C overnight. The overnight culture was used to inoculate three 2L bottles, each containing 1L TB supplemented with 8g/L glycerol, 50μg/mL kanamycin and approximately 200μL 204 Antifoam A6426 (Sigma). The culture was at 37°C until the OD600 reached ∼1.0. The culture was down-tempered to 18°C over a period of 1 h before target expression was induced by addition of 0.5mM Isopropyl β-d-1-thiogalactopyranoside (IPTG). Expression was allowed to continue overnight and cells were harvested the following morning by centrifugation (4,430g, 10 min, 4°C). The resulting cell pellet was stored at −80°C.

Cells were resuspended in IMAC wash1 buffer (50mM HEPES pH 7.6, 500mM NaCl, 0.5mM TCEP), supplemented with 6000 U Benzonase (Merck) and three tablets of Complete EDTA-free protease inhibitor (Roche Applied Science) and lysed by sonication. The cell suspension HisTrap 5mL column was equilibrated with IMAC wash1 buffer, and the size exclusion column (HiLoad 16/60 Superdex 75 Prep Grade (GE Healthcare)) was equilibrated with GF buffer (20mM HEPES pH 7.6, 500mM NaCl, 0.5mM TCEP). Purification of the protein was performed on an AKTA prime system (GE Healthcare). The filtered lysate was loaded onto the HisTrap column and washed with IMAC wash1 buffer. Bound protein was eluted from the IMAC column with a gradient increase in the imidazole concentration. The eluted protein was injected into a size exclusion column and fractions containing the target protein were identified by SDS-PAGE. Fresh TCEP was added to a final concentration of 2 mM. The protein was concentrated using a Vivaspin 20 centrifugal filter device (10,000 MWCO; Vivascience) and the identity of the protein was confirmed by mass spectrometry.

#### Electrophoretic mobility shift assay (EMSA)

For 5′ end labeling of DNA and RNA substrates with ^32^P, 10μM of RNA or DNA was incubated with T4 polynucleotide kinase (T4 PNK, NEB) in T4 PNK buffer (NEB) and incubated for 1 h at 37°C. After reaction completion, 30μL of H_2_O was added to samples to reach a final volume of 50μL, and reactions were ran through a G25 spin column (VWR) to remove free nucleotides. For single stranded substrates, 50μL of water was added to the sample to reach a final concentration of RNA/DNA of 100nM. For hairpin or double stranded substrates, 40μL of water and 10μL of 10X annealing buffer (50mM HEPES pH7.5, 50mM NaCl) was added for a final concentration of 100nM. RNA and DNA substrates were annealed at a concentration of 100nM in 1X annealing buffer by heating at 98°C for 3 min, followed by slowly cooling to room temperature.

EMSA assays were performed to assess the binding of protein to RNA and DNA substrates. 10nM of radiolabeled RNA or DNA substrate was incubated with increasing concentrations of recombinant protein (Full length TIRR purified as above, or 53BP1-Tudor (Cayman, 14073)) as indicated in 1X EMSA buffer (50mM HEPES, 50mM NaCl, 5% Glycerol, 20mM MgCl_2_, 0.01% Tween 20). Reactions were carried out in a final volume of 10μL. Reactions were incubated for 15 min at 37°C, before adding native agarose gel loading solution (Thermo Fisher) to 1X final. Reactions were run on 0.5X TBE 6% native polyacrylamide gels in 0.5X TBE running buffer (Life Technologies) for 3 h at 100V. Gels were then dried on Whatman paper for 2 h at 80°C, and imaged using autoradiography film overnight at −80°C.

#### Surface Plasmon Resonance (SPR)

Corresponding RNA or DNA substrates were annealed 95°C for 5 min in annealing buffer (10mM Tris pH 7.5, 50 mM NaCl, 1 mM EDTA) and slowly cooled to RT. All experiments were conducted on a Biacore 8K + Device and were performed at 25°C. *N*_*α*_*,N*_*α*_-Bis(carboxymethyl)-L-lysine hydrate reagent (Sigma) dissolved in 50 mM borate buffer (pH 8.5) was chemically bound to the SPR CM4 sensorchip (Cytiva, BR100534) using an amine coupling kit (Cytiva, BR100050). 6–211 TIRR-His (0.1 μM) was injected via NTA-Capture coupling on the SPR surface during 200s at 5 μL/min to reach ∼600 RU of protein immobilised (running buffer for immobilisation: PBS-P (Cytiva, 28995084)). After equilibration of the Biacore system in running buffer (HEPES 20 mM pH 6, NaCl 300 mM, p20 0.05%), DNA and RNA substrates were injected in multi-cycle kinetics over TIRR-His protein in a dose-response up to 200μM with 100s association and 100s dissociation at 30 μL/min. Binding curves were analyzed using Insight Evaluation Software and fitted when possible with a 1:1 steady-state affinity model.

#### Homogeneous Time Resolved Fluorescence (HTRF)

1536 well plates (Corning) were thawed at RT and centrifuged at 1800rpm at RT and placed on a rotating Cytomat hotel of a robotic platform. Solutions of proteins (HA-TEV-53BP1 1484–1603 and 3xFlag-TIRR, 10nM each final) and of antibodies (Cryptate donor Anti-Flag TB3+, Cisbio, 61FG2TL, 0.13μg/mL final and Cryptate acceptor Anti-HA XL665, Cisbio, 610HAXL, 2μg/mL final) were prepared in PPI buffer (Terbium detection buffer, Cisbio, 61DB10RDF). An EL406 (Biotek) was used to dispense solutions of proteins into 1536 well plates and incubated at RT for 15 min. Antibodies were then added and left to incubate for 2 h at RT. PPI buffer alone was used to measure background signal. Readings were then taken on a Tecan Spark:

Measurement 1

Excitation filter: 340(20)nm/Emission filter: 620(10)nm/MirrorDichroic: 510

Lag time: 100μs/Integration time: 300μs/Number of reads: 3/Gain: 170/Z-Position: 20500 [μm] Measurement 2

Excitation filter: 340(20)nm/Emission filter: 665(8)nm/MirrorDichroic: 510

Lag time: 100μs/Integration time: 300μs/Number of reads: 3/Gain: 160/Z-Position: 20500 [μm]

Raw data files are processed with a local tool (Screener Utilities) before implementation in Genedata Screener for QC validation, data pre-processing and data analysis. Raw data are uploaded into Genedata Screener and plates or wells that had technical issues are masked. Raw fluorescence of the donor (620 nm) and acceptor (665 nm) is recorded and the HTRF ratio is calculated. The HTRF ratio is normalised on each plate independently using the median of control wells: 100% inhibition (no target proteins in DMSO) and 0% inhibition (proteins with HTRF antibodies in DMSO).

#### Modeling and molecular dynamics simulations

For modeling of TIRR with RNA, active residues on TIRR (PDB: 6D0L (Botuyan et al., 2018)) were set by comparison with NUDT16 bound to IMP (PDB: 3COU). In PyMol(Schrodinger LLC, 2021), residues on NUDT16 within 5Å of IMP were selected as active residues and confirmed by modeling of NUDT16 and IMP in HADDOCK2.4 (van Zundert and Bonvin, 2014) with these residues. NUDT16 and TIRR were overlayed in PyMOL and the corresponding residues on NUDT16 were selected on TIRR. Residues on TIRR which did not align well with active residues on NUDT16 were excluded. HADDOCK2.4 was run for TIRR-RNA modeling, the protein-RNA method was run with ‘Easy’ mode default settings. The top three clusters with the lowest HADDOCK score were inspected manually in PyMOL and the first model was chosen. During the inspection of the model, we increased the interaction between the monomer protein and the RNA by reducing the distance between the target residues. We then used this model as the starting structure for MD simulations of the monomer in complex with the RNA. The system of the hairpin RNA bound to the TIRR dimer was built using the TIRR structure (PDB:6D0L). The information on the positions of the missing residues of the TIRR protein in the 6D0L structure were taken from the PDB structure 6CO1, and placed by overlapping the two structures using PyMOL. Both systems, RNA-TIRR monomer and RNA-TIRR dimer, were placed at the center of a truncated octahedral box of TIP3P (Jorgensen et al., 1983) water molecules leaving 1.5nm between the protein-RNA atoms and the edges of the box, neutralised by K+ ions, and additional ions (K+ and Cl) (Smith and Dang, 1994) added to reach a concentration of 150mM. Each system was minimised using a multi-step procedure (Ivani et al., 2016; Pasi et al., 2014), thermalised to 298K (in the NVT ensemble), and then equilibrated and simulated without restraints collecting 500ns for each simulation as previously described (Ivani et al., 2016; Pasi et al., 2014) in the NPT ensemble (pressure = 1atm, integration step = 2fs), using Particle-Mesh Ewald corrections (Darden et al., 1993) and periodic boundary conditions. SHAKE was used to constrain bonds involving hydrogen (Ryckaert et al., 1977). We used the revised force field parmbsc0-OL3 for the RNA (Pérez et al., 2007; Zgarbová et al., 2011) and the amber14sb force field for the protein. Each simulation was extended for at least 500ns of production time using AMBER 20 (Case et al., 2021), followed by analysis with the CPPTRAJ (Roe and Cheatham, 2013) and BIGNASIM (Hospital et al., 2016) suite of programs for the base-pair parameters. The systems were visualised with VMD v1.9.4 (Humphrey et al., 1996). The simulations are stored and available in the database BIGNASIM (Hospital et al., 2016). The electrostatic interactions between the protein residues and the RNA were determined by solving the linear Poisson–Boltzmann equation, while the van der Waals contribution was determined using standard AMBER Lennard–Jones parameters (cMIP (Gelpí et al., 2001)). The RNA structures were generated using RNAfold (Lorenz et al., 2011) and RNAComposer (Biesiada et al., 2016). For the HADDOCK model of the hairpin RNA and TIRR, the following active residues were set in HADDOCK2.4; using 6D0L chain A for TIRR (27, 29, 55, 56, 57, 61, 62, 110, 166, 168, 169, 172) and RNA hairpin (active (in loop): 21, 22, 23, 24, 25, 26).

### Quantification and statistical analysis

Statistical analysis was performed using GraphPad Prism 9. The Kolmogorov-Smirnov normality test was performed to test for a normal distribution. If data met the requirements of a normal distribution, an unpaired t test was performed. If data did not show a normal distribution, for a comparison of two groups, a Mann-Whitney test was performed. For comparison of more than two groups, a Kruskal-Wallis with Dunn’s multiple comparisons test was performed. p > 0.05 = ns, p ≤ 0.05 = ^∗^, p ≤ 0.01 = ^∗∗^, p ≤ 0.001 = ^∗∗∗^, p ≤ 0.0001 = ^∗∗∗∗^.

## Data Availability

• Data reported in this paper can be shared by the lead contact upon request. • This paper does not report original code. The MD simulations are stored and available in the database BIGNASIM https://mmb.irbbarcelona.org/BIGNASim/. • Any additional information required to reanalyze the data reported in this paper is available from the lead contact upon request.

## Supplemental information

Document S1. Figures S1–S10 and Table S1

Document S2. Article plus supplemental information

## References

[bib1] Alagia A., Ketley R.F., Gullerova M., Aguilera A., Ruzov A. (2022). R-Loops.

[bib2] Avolio R., Järvelin A.I., Mohammed S., Agliarulo I., Condelli V., Zoppoli P., Calice G., Sarnataro D., Bechara E., Tartaglia G.G. (2018). Protein Syndesmos is a novel RNA-binding protein that regulates primary cilia formation. Nucleic Acids Res..

[bib3] Bader A.S., Bushell M. (2020). DNA:RNA hybrids form at DNA double-strand breaks in transcriptionally active loci. Cell Death Dis..

[bib4] Biesiada M., Pachulska-Wieczorek K., Adamiak R.W., Purzycka K.J. (2016). RNAComposer and RNA 3D structure prediction for nanotechnology. Methods.

[bib5] Bonath F., Domingo-Prim J., Tarbier M., Friedländer M.R., Visa N. (2018). Next-generation sequencing reveals two populations of damage-induced small RNAs at endogenous DNA double-strand breaks. Nucleic Acids Res..

[bib6] Botuyan M.V., Cui G., Drané P., Oliveira C., Detappe A., Brault M.E., Parnandi N., Chaubey S., Thompson J.R., Bragantini B. (2018). Mechanism of 53BP1 activity regulation by RNA-binding TIRR and a designer protein. Nat. Struct. Mol. Biol..

[bib7] Burger K., Schlackow M., Potts M., Hester S., Mohammed S., Gullerova M. (2017). Nuclear phosphorylated Dicer processes double-stranded RNA in response to DNA damage. J. Cell Biol..

[bib8] Burger K., Schlackow M., Gullerova M. (2019). Tyrosine kinase c-Abl couples RNA polymerase II transcription to DNA double-strand breaks. Nucleic Acids Res..

[bib9] Carpenter A.E., Jones T.R., Lamprecht M.R., Clarke C., Kang I.H., Friman O., Guertin D.A., Chang J.H., Lindquist R.A., Moffat J. (2006). CellProfiler: image analysis software for identifying and quantifying cell phenotypes. Genome Biol..

[bib10] Case D.A., Metin Aktulga H., Belfon K., Ben-Shalom I.Y., Brozell S.R., Cerutti D.S., Cheatham T.E., Cruzeiro V.W.D., Darden T.A., Duke R.E. (2021).

[bib11] Chen L., Chen J.-Y., Zhang X., Gu Y., Xiao R., Shao C., Tang P., Qian H., Luo D., Li H. (2017). R-ChIP using inactive RNase H reveals dynamic coupling of R-loops with transcriptional pausing at gene promoters. Mol. Cell.

[bib12] Chen H., Yang H., Zhu X., Yadav T., Ouyang J., Truesdell S.S., Tan J., Wang Y., Duan M., Wei L. (2020). m5C modification of mRNA serves a DNA damage code to promote homologous recombination. Nat. Commun..

[bib13] Dai Y., Zhang A., Shan S., Gong Z., Zhou Z. (2018). Structural basis for recognition of 53BP1 tandem Tudor domain by TIRR. Nat. Commun..

[bib14] D’Alessandro G., Whelan D.R., Howard S.M., Vitelli V., Renaudin X., Adamowicz M., Iannelli F., Jones-Weinert C.W., Lee M., Matti V. (2018). BRCA2 controls DNA:RNA hybrid level at DSBs by mediating RNase H2 recruitment. Nat. Commun..

[bib15] Darden T., York D., Pedersen L. (1993). Particle mesh Ewald: an *N* ⋅log(*N*) method for Ewald sums in large systems. J. Chem. Phys..

[bib16] Domingo-Prim J., Endara-Coll M., Bonath F., Jimeno S., Prados-Carvajal R., Friedländer M.R., Huertas P., Visa N. (2019). EXOSC10 is required for RPA assembly and controlled DNA end resection at DNA double-strand breaks. Nat. Commun..

[bib17] Domingo-Prim J., Bonath F., Visa N. (2020). RNA at DNA double-strand breaks: the challenge of dealing with DNA:RNA hybrids. Bioessays.

[bib18] Drané P., Brault M.-E., Cui G., Meghani K., Chaubey S., Detappe A., Parnandi N., He Y., Zheng X.-F., Botuyan M.V. (2017). TIRR regulates 53BP1 by masking its histone methyl-lysine binding function. Nature.

[bib19] Francia S., Michelini F., Saxena A., Tang D., de Hoon M., Anelli V., Mione M., Carninci P., d’Adda di Fagagna F. (2012). Site-specific DICER and DROSHA RNA products control the DNA-damage response. Nature.

[bib20] Francia S., Cabrini M., Matti V., Oldani A., d’Adda di Fagagna F. (2016). DICER, DROSHA and DNA damage response RNAs are necessary for the secondary recruitment of DNA damage response factors. J. Cell Sci..

[bib21] Gelpí J.L., Kalko S.G., Barril X., Cirera J., de la Cruz X., Luque F.J., Orozco M. (2001). Classical molecular interaction potentials: improved setup procedure in molecular dynamics simulations of proteins: CMIP in the Setup of MD Simulations. Proteins.

[bib22] He C., Sidoli S., Warneford-Thomson R., Tatomer D.C., Wilusz J.E., Garcia B.A., Bonasio R. (2016). High-resolution mapping of RNA-binding regions in the nuclear proteome of embryonic stem cells. Mol. Cell.

[bib23] Hospital A., Andrio P., Cugnasco C., Codo L., Becerra Y., Dans P.D., Battistini F., Torres J., Goñi R., Orozco M. (2016). BIGNASim: a NoSQL database structure and analysis portal for nucleic acids simulation data. Nucleic Acids Res..

[bib24] Humphrey W., Dalke A., Schulten K. (1996). VMD: visual molecular dynamics. J. Mol. Graph..

[bib25] Ivani I., Dans P.D., Noy A., Pérez A., Faustino I., Hospital A., Walther J., Andrio P., Goñi R., Balaceanu A. (2016). Parmbsc1: a refined force field for DNA simulations. Nat. Methods.

[bib26] Jackson S.P., Bartek J. (2009). The DNA-damage response in human biology and disease. Nature.

[bib27] Jimeno S., Balestra F.R., Huertas P. (2021). The emerging role of RNA modifications in DNA double-strand break repair. Front. Mol. Biosci..

[bib28] Jorgensen W.L., Chandrasekhar J., Madura J.D., Impey R.W., Klein M.L. (1983). Comparison of simple potential functions for simulating liquid water. J. Chem. Phys..

[bib29] Ketley R.F., Gullerova M. (2020). Jack of all trades? The versatility of RNA in DNA double-strand break repair. Essays Biochem..

[bib30] Liu S., Hua Y., Wang J., Li L., Yuan J., Zhang B., Wang Z., Ji J., Kong D. (2021). RNA polymerase III is required for the repair of DNA double-strand breaks by homologous recombination. Cell.

[bib31] Lorenz R., Bernhart S.H., Höner zu Siederdissen C., Tafer H., Flamm C., Stadler P.F., Hofacker I.L. (2011). ViennaRNA package 2.0. Algorithms Mol. Biol..

[bib32] Marnef A., Legube G. (2021). R-loops as Janus-faced modulators of DNA repair. Nat. Cell Biol..

[bib33] Massip L., Caron P., Iacovoni J.S., Trouche D., Legube G. (2010). Deciphering the chromatin landscape induced around DNA double strand breaks. Cell Cycle.

[bib34] Mhlanga M.M., Vargas D.Y., Fung C.W., Kramer F.R., Tyagi S. (2005). tRNA-linked molecular beacons for imaging mRNAs in the cytoplasm of living cells. Nucleic Acids Res..

[bib35] Michelini F., Pitchiaya S., Vitelli V., Sharma S., Gioia U., Pessina F., Cabrini M., Wang Y., Capozzo I., Iannelli F. (2017). Damage-induced lncRNAs control the DNA damage response through interaction with DDRNAs at individual double-strand breaks. Nat. Cell Biol..

[bib36] Noordermeer S.M., van Attikum H. (2019). PARP inhibitor resistance: a tug-of-war in BRCA-mutated cells. Trends Cell Biol..

[bib37] Panier S., Boulton S.J. (2014). Double-strand break repair: 53BP1 comes into focus. Nat. Rev. Mol. Cell Biol..

[bib38] Pasi M., Maddocks J.H., Beveridge D., Bishop T.C., Case D.A., Cheatham T., Dans P.D., Jayaram B., Lankas F., Laughton C. (2014). μABC: a systematic microsecond molecular dynamics study of tetranucleotide sequence effects in B-DNA. Nucleic Acids Res..

[bib39] Pérez A., Marchán I., Svozil D., Sponer J., Cheatham T.E., Laughton C.A., Orozco M. (2007). Refinement of the AMBER force field for nucleic acids: improving the description of α/γ conformers. Biophys. J..

[bib40] Pessina F., Giavazzi F., Yin Y., Gioia U., Vitelli V., Galbiati A., Barozzi S., Garre M., Oldani A., Flaus A. (2019). Functional transcription promoters at DNA double-strand breaks mediate RNA-driven phase separation of damage-response factors. Nat. Cell Biol..

[bib41] Rassoolzadeh H., Coucoravas C., Farnebo M. (2015). The proximity ligation assay reveals that at DNA double-strand breaks WRAP53β associates with γH2AX and controls interactions between RNF8 and MDC1. Nucleus.

[bib42] Roe D.R., Cheatham T.E. (2013). PTRAJ and CPPTRAJ: software for processing and analysis of molecular dynamics trajectory data. J. Chem. Theory Comput..

[bib43] Ryckaert J.-P., Ciccotti G., Berendsen H.J.C. (1977). Numerical integration of the cartesian equations of motion of a system with constraints: molecular dynamics of n-alkanes. J. Comput. Phys..

[bib44] Schindelin J., Arganda-Carreras I., Frise E., Kaynig V., Longair M., Pietzsch T., Preibisch S., Rueden C., Saalfeld S., Schmid B. (2012). Fiji: an open-source platform for biological-image analysis. Nat. Methods.

[bib45] Schrodinger LLC (2021).

[bib46] Smith D.E., Dang L.X. (1994). Computer simulations of NaCl association in polarizable water. J. Chem. Phys..

[bib47] Vítor A.C., Sridhara S.C., Sabino J.C., Afonso A.I., Grosso A.R., Martin R.M., de Almeida S.F. (2019). Single-molecule imaging of transcription at damaged chromatin. Sci. Adv..

[bib48] Wang J., Yuan Z., Cui Y., Xie R., Yang G., Kassab M.A., Wang M., Ma Y., Wu C., Yu X. (2018). Molecular basis for the inhibition of the methyl-lysine binding function of 53BP1 by TIRR. Nat. Commun..

[bib49] Wei W., Ba Z., Gao M., Wu Y., Ma Y., Amiard S., White C.I., Rendtlew Danielsen J.M., Yang Y.-G., Qi Y. (2012). A role for small RNAs in DNA double-strand break repair. Cell.

[bib50] Zgarbová M., Otyepka M., Šponer J., Mládek A., Banáš P., Cheatham T.E., Jurečka P. (2011). Refinement of the Cornell et al. Nucleic Acids Force Field Based on Reference Quantum Chemical Calculations of Glycosidic Torsion Profiles. J. Chem. Theory Comput..

[bib51] Zhang A., Peng B., Huang P., Chen J., Gong Z. (2017). The p53-binding protein 1-Tudor-interacting repair regulator complex participates in the DNA damage response. J. Biol. Chem..

[bib52] Zhang C., Chen L., Peng D., Jiang A., He Y., Zeng Y., Xie C., Zhou H., Luo X., Liu H. (2020). METTL3 and N6-methyladenosine promote homologous recombination-mediated repair of DSBs by modulating DNA-RNA hybrid accumulation. Mol. Cell.

[bib53] van Zundert G.C.P., Bonvin A.M.J.J. (2014). Modeling protein–protein complexes using the HADDOCK webserver “modeling protein complexes with HADDOCK. Methods Mol. Biol..

