# Supplementary material for: DNA double-strand break-derived RNA drives TIRR/53BP1 complex dissociation
Source: Cell Rep. 2022 Oct 25;41(4):111526. doi: 10.1016/j.celrep.2022.111526 (PMC9638026; doi:10.1016/j.celrep.2022.111526)
Supplement: Document S1. Figures S1–S10 and Table S1 [file mmc1.pdf]

Cell Reports, Volume *41*

Supplemental information

DNA double-strand break-derived RNA drives TIRR/53BP1 complex dissociation

Ruth F. Ketley, Federica Battistini, Adele Alagia, Clémence Mondielli, Florence Iehl, Esra Balikçi, Kilian V.M. Huber, Modesto Orozco, and Monika Gullerova

Supplementary Figure 1

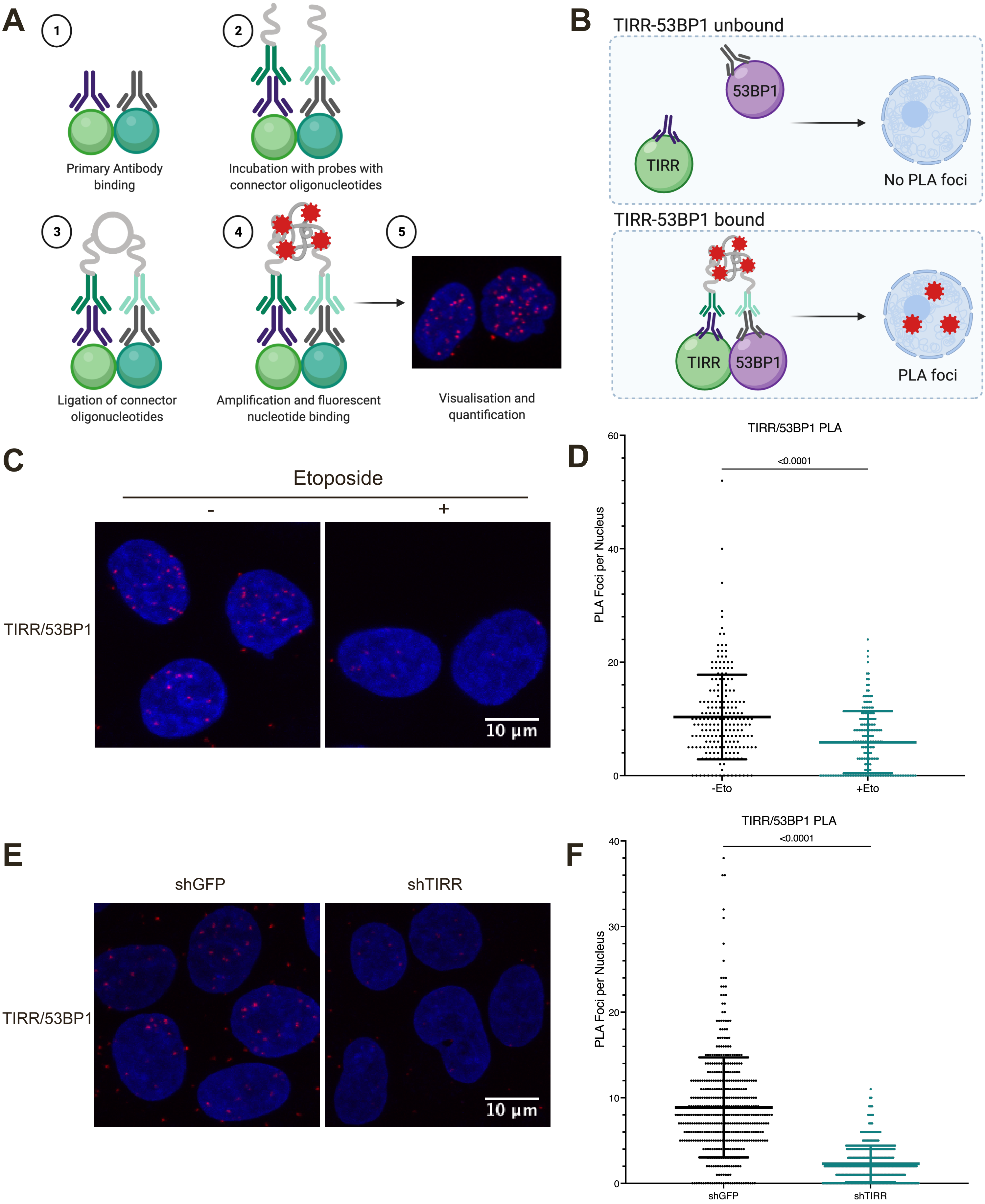

Supplementary Figure 2

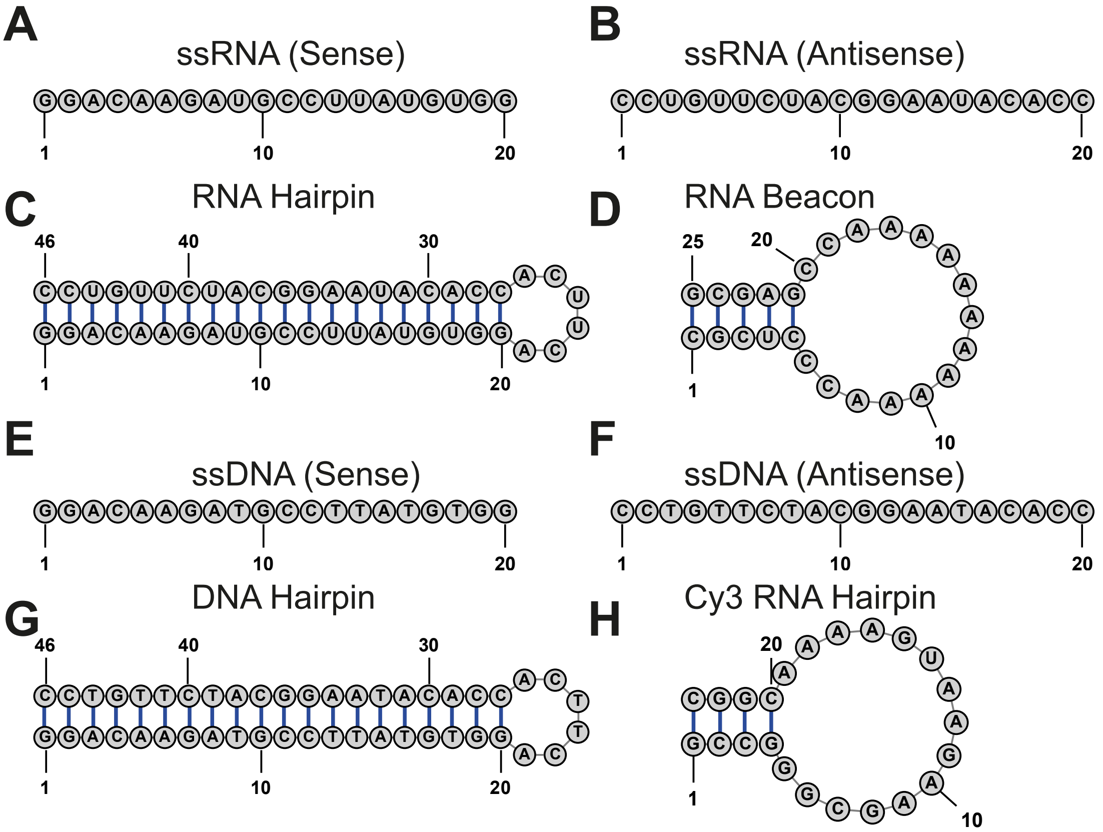

Supplementary Figure 3

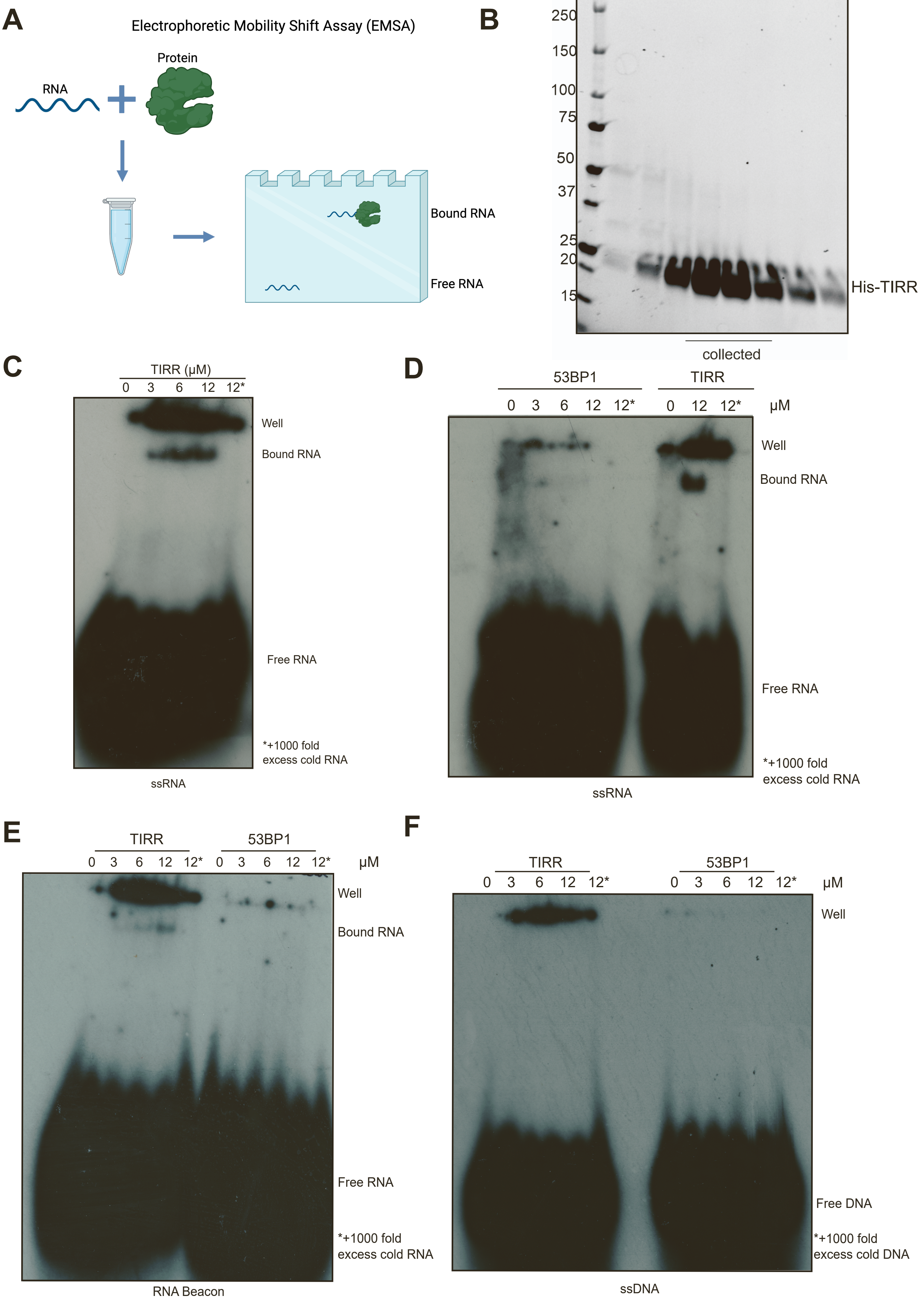

Supplementary Figure 4

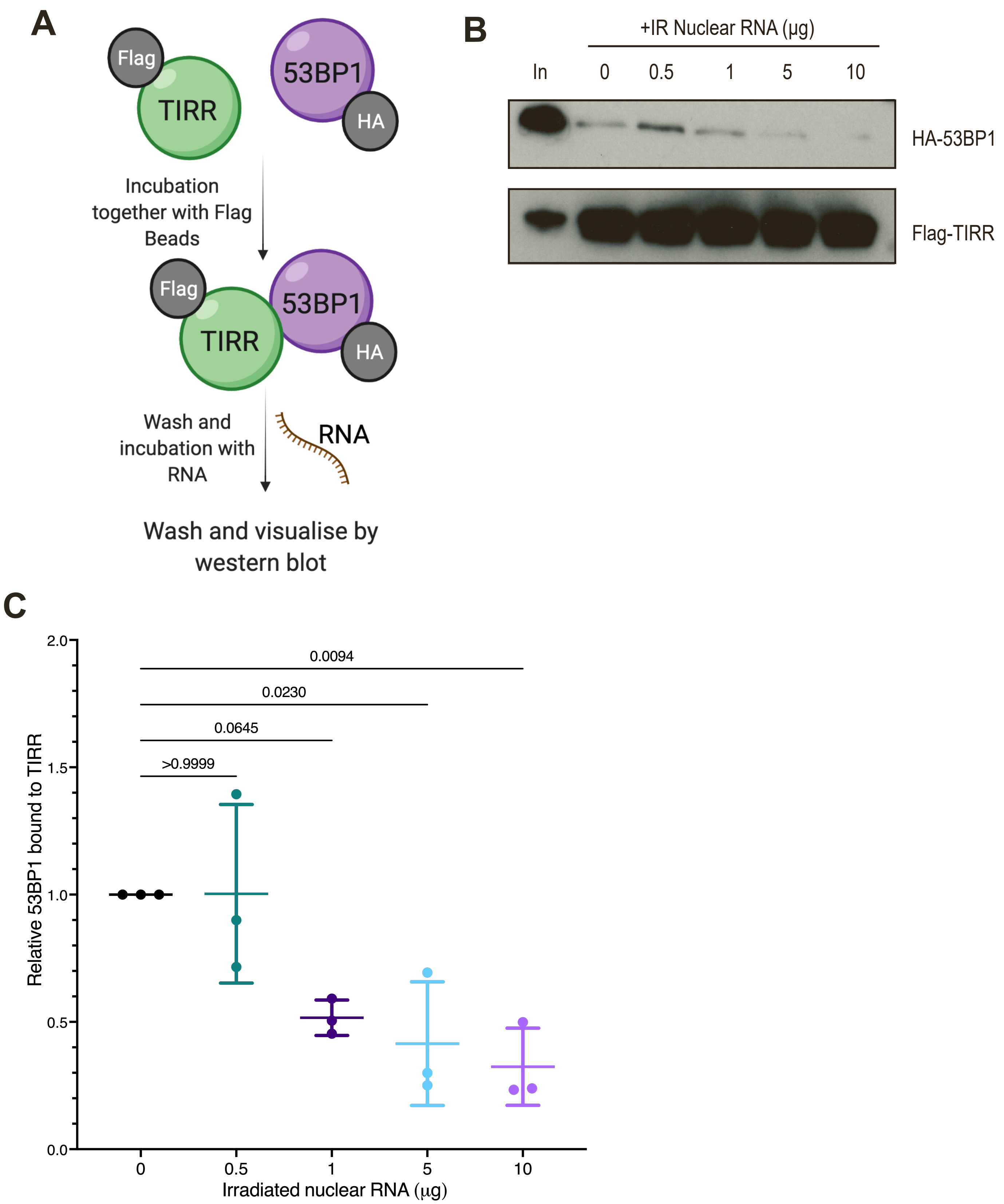

Supplementary Figure 5

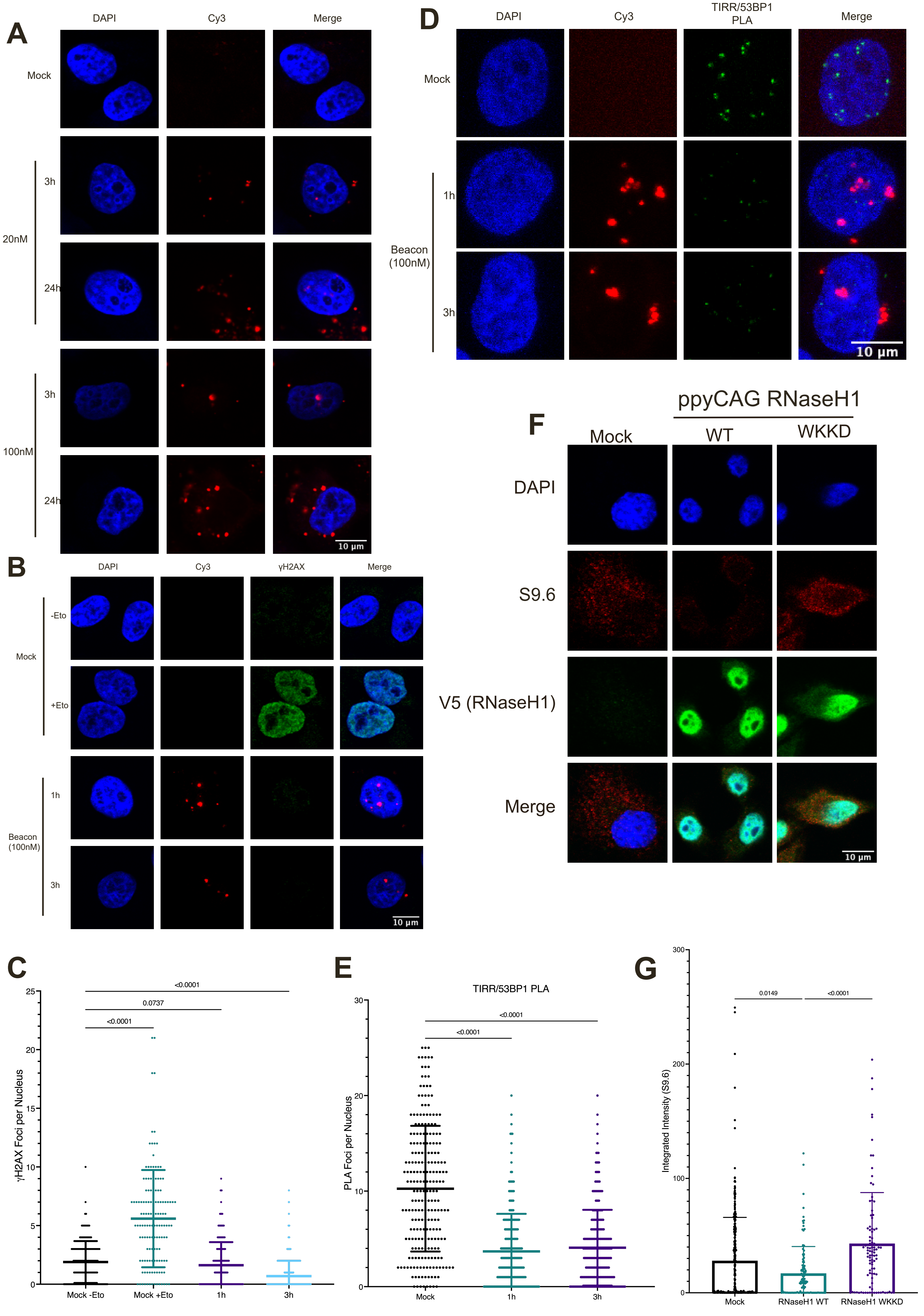

Supplementary Figure 6

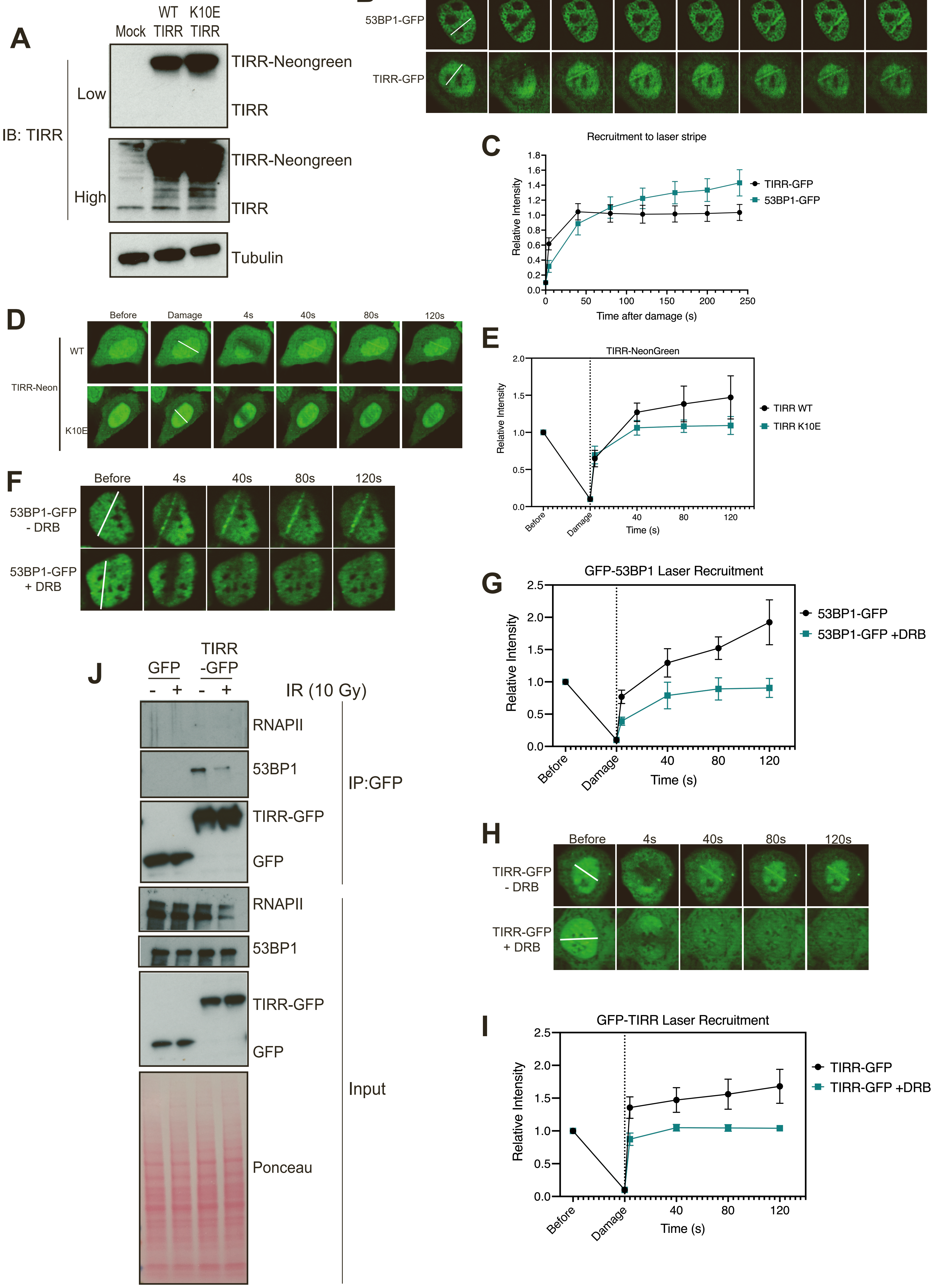

Supplementary Figure 7

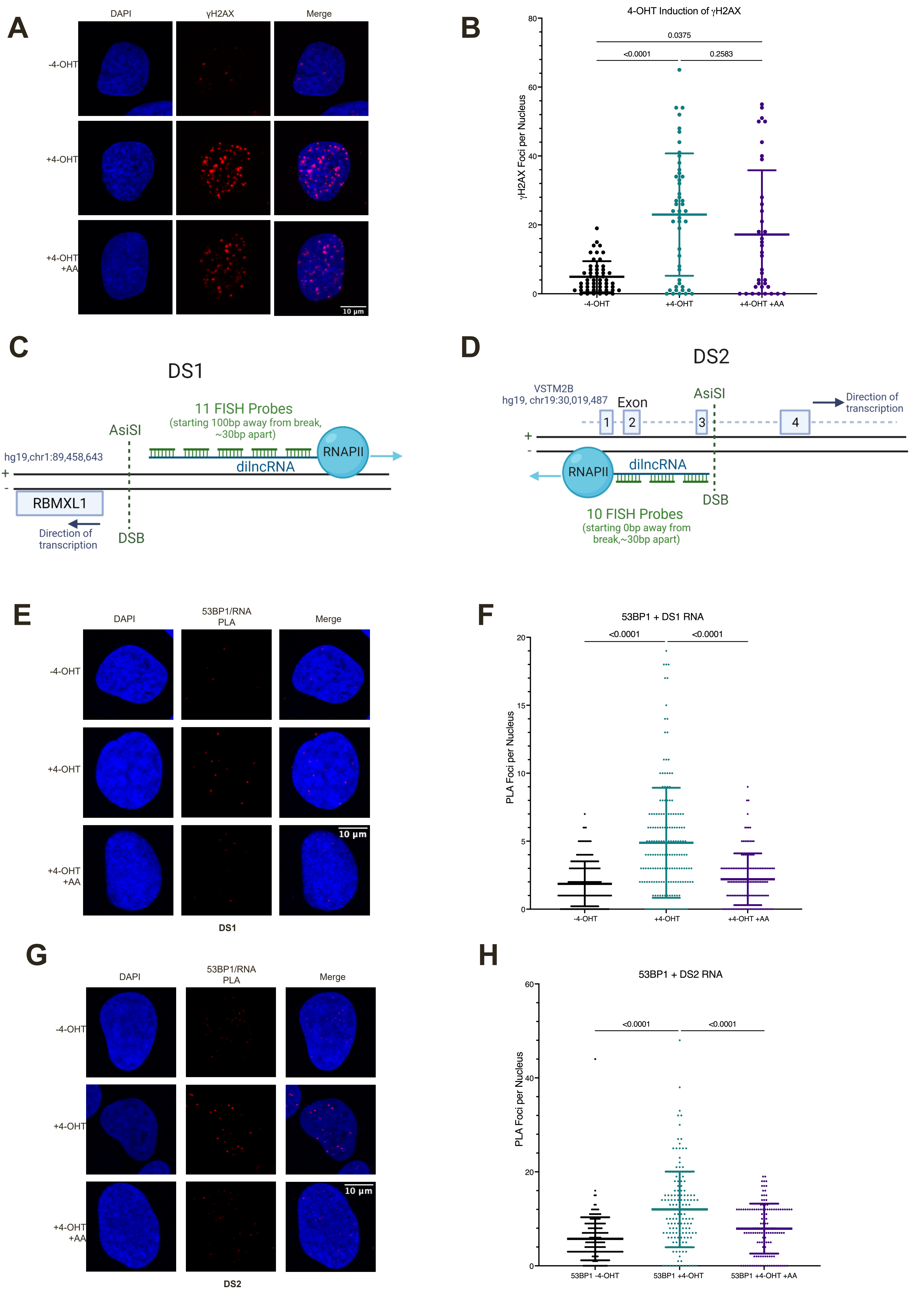

Supplementary Figure 8

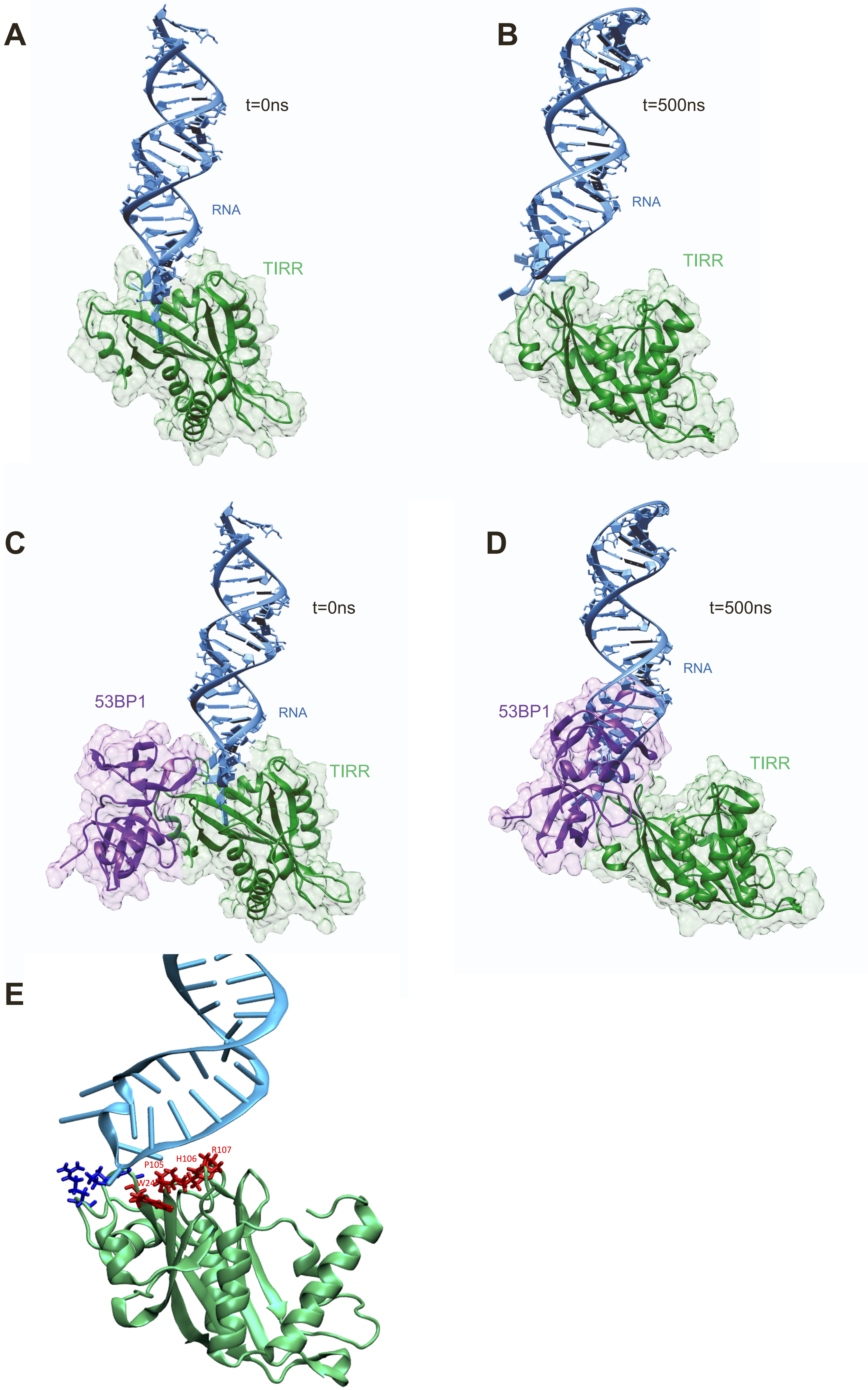

Supplementary Figure 9

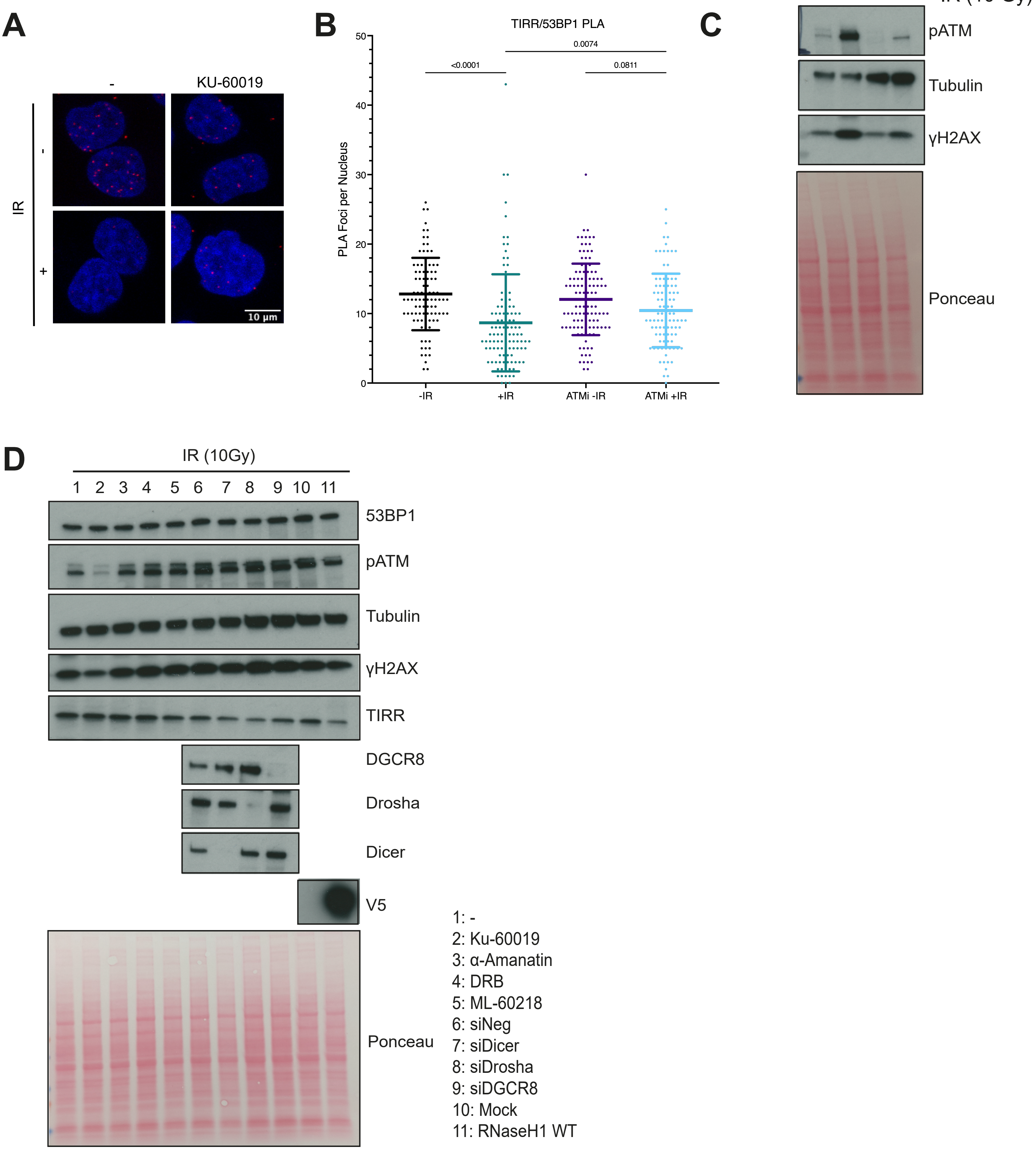

1: -
2: Ku-60019
3: α-Amanatin
4: DRB
5: ML-60218
6: siNeg
7: siDicer
8: siDrosha
9: siDGCR8
10: Mock
11: RNaseH1 WT

Supplementary Figure 10

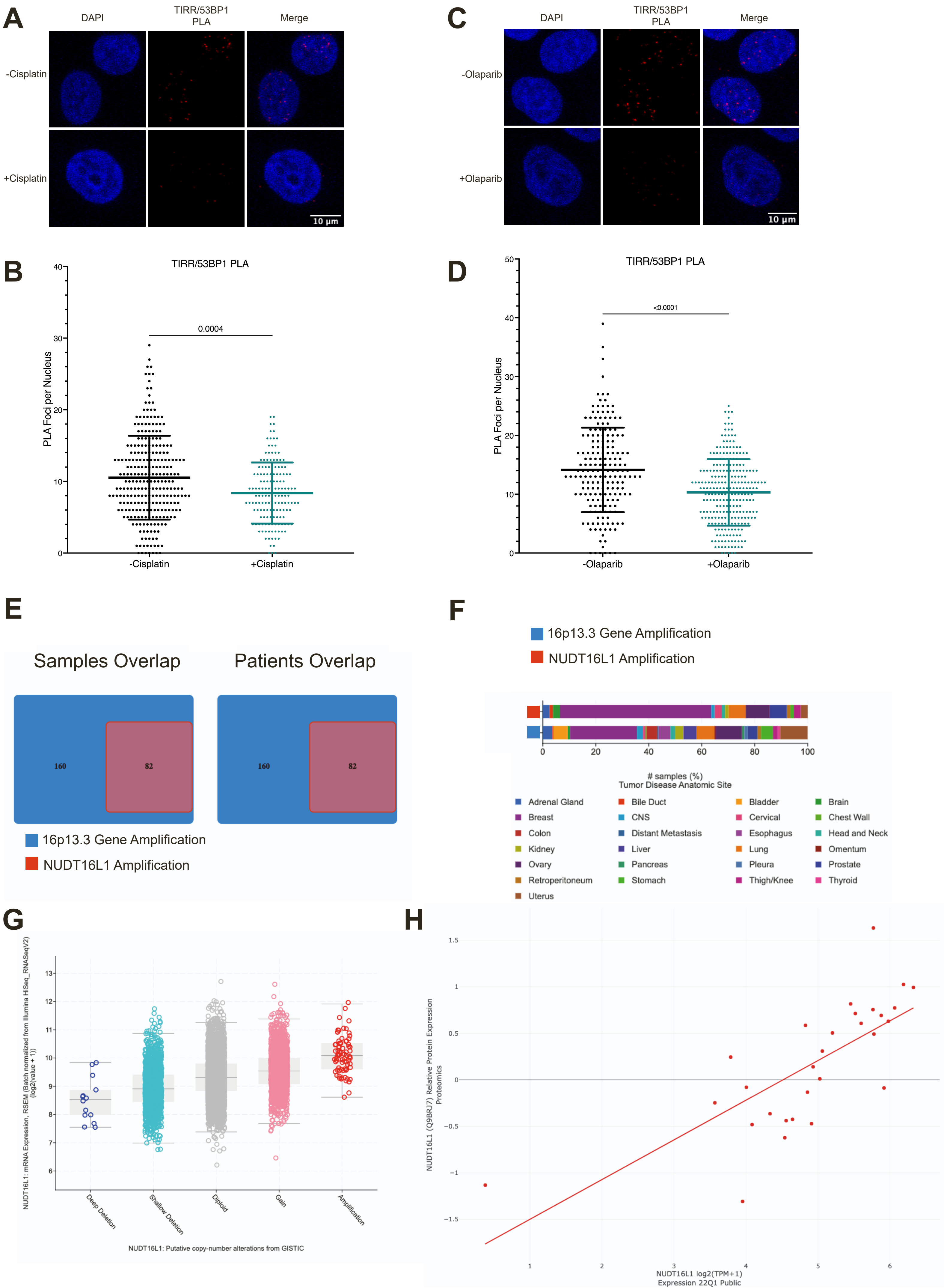

Supplemental information

Probe	Sequence (5' → 3')
FISH_PLA_DS1_1	CCGGCCGACTCCACAGCCAATGGAGTTCCCTGAGTCCACACCCATAGCCAAAAAAAAAAAAAAAAAAAAAAAAAAAAAAAAAAAAAAAAATATGACAGAACTAGACAC
FISH_PLA_DS1_2	CCAAATTCCATACCCATATCCAAGTCCCCGCCCCACTAACTCGAGCCCGCAAAAAAAAAAAAAAAAAAAAAAAAAAAAAAAAAAAAAAATATGACAGAACTAGACAC
FISH_PLA_DS1_3	GCCCATCCTTGCAAACCAGTCCTCGTCCTATAACCTGCCCCAACCCGATCAAAAAAAAAAAAAAAAAAAAAAAAAAAAAAAAAAAAAAAATATGACAGAACTAGACAC
FISH_PLA_DS1_4	CCCTGCCCTCTCTCAGGCACCTTCCAAAGTTATTCCAACCCGATCCCTCCAAAAAAAAAAAAAAAAAAAAAAAAAAAAAAAAAAAAAAAATATGACAGAACTAGACAC
FISH_PLA_DS1_5	GTTCCTTTACGCTTTTTAACCCCACACCCCCATCTCAAATGACAAATGCAAAAAAAAAAAAAAAAAAAAAAAAAAAAAAAAAAAAAAAATATGACAGAACTAGACAC
FISH_PLA_DS1_6	GTCATTTTTCTTCCTTCTTCAGTCTCACATGTCCCTTTCATTCTTAGTTTAAAAAAAAAAAAAAAAAAAAAAAAAAAAAAAAAAAAAAAATATGACAGAACTAGACAC
FISH_PLA_DS1_7	TTCGCTGACACTGCCCTCCCCAAAGAAGTCACCAGTGGTTTCCTTGTTTTAAAAAAAAAAAAAAAAAAAAAAAAAAAAAAAAAAAAAAAATATGACAGAACTAGACAC
FISH_PLA_DS1_8	CCCTCTAGCCTCTCATCCTCTTAAAATAGAAGAGGAAAAATATCCAAACAAAAAAAAAAAAAAAAAAAAAAAAAAAAAAAAAAAAAAAATATGACAGAACTAGACAC
FISH_PLA_DS1_9	GAAGAGTATATTCCTTTAATCAATGTATACTTTTGTTCCCTCCTTTAGAAAAAAAAAAAAAAAAAAAAAAAAAAAAAAAAAAAAAAAAAAATATGACAGAACTAGACAC
FISH_PLA_DS1_10	CACATCCAGGCTCGCCCCTACCTGTACACAATAATTTCTCCATGCACCTAAAAAAAAAAAAAAAAAAAAAAAAAAAAAAAAAAAAAAAAATATGACAGAACTAGACAC
FISH_PLA_DS1_11	TGAGATGTAGTATGATCACCAATCGCGGGGGACCCCACAGCCAGTGCGCGAAAAAAAAAAAAAAAAAAAAAAAAAAAAAAAAAAAAAAATATGACAGAACTAGACAC
DS2_VSTM2B_FISH_1F	AGGATGCAACTAAAATCAGCGTAAGTGTGGAGCCCAGCGCGGGCCGCGGGAAAAAAAAAAAAAAAAAAAAAAAAAAAAAAAAAAAAAAAAATATGACAGAACTAGACAC
DS2_VSTM2B_FISH_2F	CCTTGCCTAAAGGCGGATCCGAGTTCCCCTAGCCAGAAGGCCGCGAGCCTAAAAAAAAAAAAAAAAAAAAAAAAAAAAAAAAAAAAAAAAAATATGACAGAACTAGACAC
DS2_VSTM2B_FISH_3F	CTGGGAAACTCTTGGAAAGCCGGACGTCCTTTGTGCCCTCAACCCCCATCAAAAAAAAAAAAAAAAAAAAAAAAAAAAAAAAAAAAAAAATATGACAGAACTAGACAC
DS2_VSTM2B_FISH_4F	CATCCAGTCTCCCCAGTTCAGCCGGCAGGTGTGCACCAGGGCAGCCACCCAAAAAAAAAAAAAAAAAAAAAAAAAAAAAAAAAAAAAAAAATATGACAGAACTAGACAC
DS2_VSTM2B_FISH_5F	GTCCAGACACAGCGGCCCTCCCTCCAGTTCTTCACGGCTCCCAGTGAGCTAAAAAAAAAAAAAAAAAAAAAAAAAAAAAAAAAAAAAAATATGACAGAACTAGACAC
DS2_VSTM2B_FISH_6F	GTCTGCCTCAAAACCCACACTCTCTTACACACTCTAGTGTGCTCGCGTGCAAAAAAAAAAAAAAAAAAAAAAAAAAAAAAAAAAAAAAAATATGACAGAACTAGACAC
DS2_VSTM2B_FISH_7F	GCCACCAGAAAACCGAGTACCGGAAAGCCGGCAGGACCTCCAGCCCTCAGAAAAAAAAAAAAAAAAAAAAAAAAAAAAAAAAAAAAAAAAATATGACAGAACTAGACAC
DS2_VSTM2B_FISH_8F	TGCTCGCTGGCTGCGGCCCTCTGATGGGTGCTGGGTAGTGAAGGAAGCCCAAAAAAAAAAAAAAAAAAAAAAAAAAAAAAAAAAAAAAAAATATGACAGAACTAGACAC
DS2_VSTM2B_FISH_9F	CAGCAGTGGTTACCCCCAAACCTGTCAATTATTTTGACAGCATCGCTGTTAAAAAAAAAAAAAAAAAAAAAAAAAAAAAAAAAAAAAAAAATATGACAGAACTAGACAC
DS2_VSTM2B_FISH10F	AGGGAGGGAAGCAGCGGGCTTCACTCGCGCAGGGCGCCGCCCTGTGGCGCAAAAAAAAAAAAAAAAAAAAAAAAAAAAAAAAAAAAAAAATATGACAGAACTAGACAC

Table S1: Sequences of FISH-PLA probes, related to STAR method FISH-PLA

Supplemental Figure Legends

Figure S1. Related to Figure 1. TIRR/53BP1 dissociate upon DNA damage
A) The principles of the Proximity Ligation Assay. Figure adapted from (Alagia et al., 2022). Image was created with Biorender.com. **B)** Depiction of TIRR and 53BP1 bound (resulting in PLA foci) and unbound (resulting in no PLA foci). Image was created with Biorender.com. **C)** PLA of TIRR and 53BP1 with Etoposide (10µM, 2 hours). n(-Eto)=206, n(+Eto)=191. **D)** Quantification of C (mean ± sd, n=2). **E)** PLA of TIRR and 53BP1 in shGFP and shTIRR inducible Flp-IN TRex Hela cells (n(shGFP)=453, n(shTIRR)=521). **F)** Quantification of E (mean ± sd, n=2).

Figure S2. Related to Figure 2. RNA and DNA Structures
RNA and DNA structures used in *in vitro* and *in vivo* experiments. **A)** ssRNA (1, Sense). **B)** ssRNA 2 (Antisense to A, annealed with A to form dsRNA). **C)** RNA Hairpin. **D)** RNA Beacon. **E)** ssDNA, annealed with ssRNA to form RNA:DNA hybrid. **F)** ssDNA (Antisense to E). **G)** DNA Hairpin. **H)** RNA hairpin labelled with Cy3 at the 5'.

Figure S3. Related to Figure 2. TIRR binds hairpin and single stranded RNA *in vitro*
A) Representation of the principles of the EMSA assay. Protein and radiolabeled RNA or DNA are incubated together and electrophoresed on a native PAGE. Free RNA/DNA migrates further through the gel, but the migration of protein bound RNA/DNA is impaired, resulting in a distinctive shift of the radioactive band detected. Image was created with Biorender.com. **B)** SDS-PAGE of purified His-TIRR fractions from a size exclusion column. Collected fractions are indication. **C)** EMSA with ssRNA with increasing TIRR concentration. **D)** EMSA with ssRNA with increasing concentration of 53BP1-tudor domain. TIRR is used as a positive control for binding. **E)** EMSA with RNA beacon and increasing concentration of TIRR and 53BP1-tudor. **F)** EMSA with ssDNA with increasing concentration of TIRR and 53BP1-tudor.

Figure S4. Related to Figure 3. RNA isolated from damaged cells promotes TIRR/53BP1 separation *in vitro*
A) Representation of *in vitro* RNA competition. Flag-TIRR and HA-53BP1 are incubated together with Flag beads, after which nuclear irradiated RNA is added. The amount of 53BP1 bound to TIRR after RNA addition is visulised by western blotting. Image was created with Biorender.com. **B)** Western blot showing amount of HA-53BP1 bound to Flag-TIRR after Flag pull down in RNA competition experiments with increasing amounts of nuclear irradiated RNA. **C)** Quantification of B (mean ± sd, n=3).

Figure S5. Related to Figure 3. Synthetic hairpin RNA is localised in the nucleus and does not induce DNA damage
A) Immunofluorescence microscopy of Hela cells transfected with a Cy3 labelled RNA hairpin at 20nM or 100nM fixed 3 hours and 24 hours post-transfection. **B)** Immunofluorescence microscopy of cells transfected with Cy3 labelled RNA hairpin and probing for ɣH2AX. Etoposide was added as a positive control for damage induction (n(Mock -Eto)=158, n(Mock +Eto)=134, n(1h)=294, n(3h)=330). **C)** Quantification of B (mean ± sd, n=2). **D)** PLA of TIRR and 53BP1 with a second Cy3 labelled RNA hairpin with a different sequence. (n(Mock)=230, n(1h)=249, n(3h)=262. **E)** Quantification of D. (mean ± sd, n=2) **F)** Immunofluorescence of S9.6 inMock, RNaseH1 WT, or RNaseH1 WKKD transfected cells to assess digestion of RNA:DNA hybrids. (n(Mock)=328, n(WT)=111, n(WKKD)=94. **G)** Quantification of F (mean ± sd, n=1).

Figure S6. Related to Figure 5. The TIRR/53BP1 complex is localised at DSBs
A) Western blot to confirm expression of WT Neongreen-TIRR and K10E Neongreen-TIRR after transfection. **B)** Laser stripping of 53BP1-GFP U2OS cells and TIRR-GFP Hela cells. The white line depicts the location of the laser stripe. **C)** Quantification of B (mean ± sd, n=1). **D)** Laser stripping of Hela cells with transfection prior to damage of either mock, WT Neongreen-TIRR, or K10E Neongreen-TIRR. The white line depicts the location of the laser stripe. **E)** Quantification of D (mean ± sd, n=1). **F)** Laser stripping of 53BP1-GFP U2OS cells with and without DRB (100µM, 1 hour) prior to damage. The white line depicts the location of the laser stripe. **G)** Quantification of F (mean ± sd, n=1). **H)** Laser striping of TIRR-GFP Hela cells with and without DRB (100µM, 1 hour) prior to damage. The white line depicts the location of the laser stripe. **I)** Quantification of H (mean ± sd, n=1). **J)** Co-IP of GFP or TIRR-GFP in non-damage or irradiated cells.

Figure S7. Related to Figure 6. The TIRR/53BP1 complex is in close proximity to RNA transcribed at the DSB
A) Induction of DSBs as measured by ɣH2AX foci after the addition of 4-OHT, with the addition of α-amanitin. n(-4-OHT)=53, n(+4-OHT)=48, n(-4-OHT +AA)=36. **B)** Quantification of A (mean ± sd, n=1). **C)** Design of FISH-PLA probes to DS1. Image was created with Biorender.com. **D)** Design of FISH-PLA probes to DS2. Image was created with Biorender.com. **E)** FISH-PLA of 53BP1 and DS1 probes, with and without 4-OHT (500nM, 4 hours), or α-amanitin (2µg/ml, 24 hours). n(-4-OHT)=169, n(+4-OHT)=171, n(-4-OHT +AA)=135. **F)** Quantification of C (mean ± sd, n=2). **G)** FISH-PLA of 53BP1 and DS2 probes, with and without 4-OHT (500nM, 4 hours), or α-amanitin (2µg/ml, 24 hours). n(-4-OHT)=187, n(+4-OHT)=161, n(-4-OHT +AA)=129. **H)** Quantification of E (mean ± sd, n=2).

Figure S8. Related to Figure 7. MD simulations of hairpin RNA with the TIRR monomer results in greater movement than with the TIRR dimer, but still occupies the 53BP1 binding site
Complex of the TIRR monomer protein (green) with the RNA hairpin (cyan) **A)** at the start of the MD simulation (after equilibration) and **B)** after 500ns of MD simulation. **C)** Complex of TIRR (green) with the RNA hairpin (cyan) overlapped with the complex of TIRR/53BP1 (purple), at the start of the MD simulation. **D)** as in C, at the end of the simulation. **E)** Complex of TIRR (green) with the RNA hairpin (cyan). Residues highlighted (licorice) in TIRR which interact with the RNA. In red are the residues known to interact and recognise 53BP1, in blue are additional residues of the TIRR monomer binding the RNA.

Figure S9. Related to Figure 1 and 4. The influence of ATM on TIRR and 53BP1 dissociation
A) PLA of TIRR and 53BP1 with ATMi (KU-60019, 10µM, 1 hour). n(-IR)=96, n(+109)=255, n(ATMi -IR)=119, n(ATMi +IR)=93. **B)** Quantification of A (mean ± sd, n=1). **C**) Western blot to assess for phosphorylated ATM levels after ATM inhibition with KU-60019. **D)** Western blot to assess for phosphorylated ATM levels after RNAPII inhibition, siRNA knockdown of Dicer, Drosha, and DGCR8, or transfection of RNaseH1 WT.

Figure S10. Related to Figure 7. TIRR and 53BP1 can be dissociated by Cisplatin and Olaparib and TIRR is frequently amplified in breast cancer as part of the 16p13.3 amplicon
A) PLA of TIRR and 53BP1 with Cisplatin (10µM, 24 hours). n(-Cisplatin)=265, n(+Cisplatin)=150. **B)** Quantification of A (mean ± sd, n=2). **C)** PLA of TIRR and 53BP1 with Olaparib (10µM, 24 hours). n(-Olaparib)=188, n(+Olaparib)=255. **D)** Quantification of C

(mean ± sd, n=2). **E)** Overlap of samples and patients with NUDT16L1/TIRR amplification and amplification of any gene which falls within the 16p13.3 region (the region where TIRR is found), without TIRR. **F)** Breakdown of cancer sites of samples with NUDT16L1/TIRR amplification and amplification of any gene which falls within the 16p13.3 region (the region where TIRR is found), without TIRR. Displayed as a percentage. **G)** RNA expression of TIRR with deletion or amplification of the TIRR gene. **H)** RNA expression vs protein expression of TIRR in breast cancer.
